# Meteorological conditions, climate change, new emerging factors, and asthma and related allergic disorders. A statement of the World Allergy Organization

**DOI:** 10.1186/s40413-015-0073-0

**Published:** 2015-07-14

**Authors:** Gennaro D’Amato, Stephen T. Holgate, Ruby Pawankar, Dennis K. Ledford, Lorenzo Cecchi, Mona Al-Ahmad, Fatma Al-Enezi, Saleh Al-Muhsen, Ignacio Ansotegui, Carlos E. Baena-Cagnani, David J. Baker, Hasan Bayram, Karl Christian Bergmann, Louis-Philippe Boulet, Jeroen T. M. Buters, Maria D’Amato, Sofia Dorsano, Jeroen Douwes, Sarah Elise Finlay, Donata Garrasi, Maximiliano Gómez, Tari Haahtela, Rabih Halwani, Youssouf Hassani, Basam Mahboub, Guy Marks, Paola Michelozzi, Marcello Montagni, Carlos Nunes, Jay Jae-Won Oh, Todor A. Popov, Jay Portnoy, Erminia Ridolo, Nelson Rosário, Menachem Rottem, Mario Sánchez-Borges, Elopy Sibanda, Juan José Sienra-Monge, Carolina Vitale, Isabella Annesi-Maesano

**Affiliations:** 1 Department of Respiratory Diseases, Division of Pneumology and Allergology, High Specialty Hospital “A. Cardarelli” Napoli, Italy, University of Naples Medical School, Via Rione Sirignano, 10, 80121 Napoli, Italy; 2 Southampton General Hospital, Clinical and Experimental Sciences, University of Southampton, Hampshire, UK; 3 Department of Pediatrics, Nippon Medical School, Tokyo, Japan; 4 Morsani College of Medicine, University of South Florida, Tampa, Florida USA; 5 Interdepartmental Centre of Bioclimatology, University of Florence Allergy and Clinical Immunology Section, Azienda Sanitaria di Prato, Italy; 6 Department of Allergy, Al-Rashid Center, Ministry of Health, Khobar, Kuwait; 7 Al-Rashid Allergy and Respiratory Center, Khobar, Kuwait; 8 Department of Pediatrics, College of Medicine, King Saud University, Riyadh, Saudi Arabia; 9 Department of Allergy and Immunology, Hospital Quirón Bizkaia, Erandio, Spain; 10 Centre for Research in Respiratory Medicine, Faculty of Medicine, Catholic University of Córdoba, Córdoba, Argentina; 11 Emeritus Consultant Anaesthesiologist, SAMU de Paris, Hôpital Necker – Enfants Malades, Paris, France; 12 Department of Chest Diseases, Respiratory Research Laboratory, Allergy Division, School of Medicine, University of Gaziantep, Şehitkamil/Gaziantep, 27310 Turkey; 13 Allergy-Centrum-Charité, Charité-Universitätsmedizin Berlin, Berlin, Germany; 14 Quebec Heart and Lung Institute, Laval University, 2725 chemin Sainte-Foy, Quebec City, G1V 4G5 Canada; 15 ZAUM – Center of Allergy and Environment, Helmholtz Zentrum München/Technische Universität München, Munich, Germany; 16 University of Naples, Institute of Respiratory Diseases, Naples, Italy; 17 World Allergy Organization, Milwaukee, Wisconsin United States; 18 Centre for Public Health Research, Massey University, Wellington, New Zealand; 19 Consultant in Emergency Medicine, Chelsea and Westminster Hospital, London, UK; 20 Development Assistance Committee, Organisation of Economic Cooperation and Development, Paris, France; 21 Asthma & Allergy Unit, Hospital San Bernardo, Salta, Argentina; 22 Skin and Allergy Hospital, Helsinki University Hospital, Helsinki, Finland; 23 Prince Naif Center for Immunology Research, College of Medicine, King Saud University, P.O.Box 2925, Postal Code 11461 Riyadh, Saudi Arabia; 24 Epidemiology of Respiratory and Allergic Disease Department, UMR-S, Institute Pierre Louis of Epidemiology and Public Health, INSERM Medical School Saint-Antoine, UPMC Sorbonne Universités, Paris, France; 25 University of Sharjah, and, Rashid Hospital DHA, Abu Dhabi, United Arab Emirates; 26 South Western Sydney Clinical School, UNSW, Australia and Woolcock Institute of Medical Research, University of Sydney, Sydney, Australia; 27 Dipartimento Epidemiologia Regione Lazio, UOC Epidemiologia Ambientale, Roma, Italy; 28 Department of Clinical and Experimental Medicine, University of Parma, Via Gramsci 14, 43100 Parma, Italy; 29 Center of Allergy of Algarve, Hospital Particular do Algarve, Particular do Algarve, Brasil; 30 Department of Pediatrics, Hanyang University College of Medicine, Seoul, Korea; 31 Clinic of Allergy and Asthma, Medical University in Sofia, Sofia, Bulgaria; 32 Children’s Mercy Hospitals & Clinics, Kansas City, Missouri USA; 33 Division of Pediatric Respiratory Medicine, Hospital de Clínicas, Federal University of Parana, Rua Tte. João Gomes da Silva 226, 80810-100 Curitiba, PR Brazil; 34 Allergy Asthma and Immunology, Emek Medical Center, Afula, and the Rappaport Faculty of Medicine Technion, Israel Institute of Technology, Haifa, Israel; 35 Clinica El Avila, Caracas, Venezuela; 36 Asthma, Allergy and Immune Dysfunction Clinic, Harare, Zimbabwe; 37 Allergy and Immunology Department, Hospital Infantil de México Federico Gómez, SSA, México City, Mexico; 38 University of Naples, Institute of Respiratory Diseases, Naples, Italy; 39 Epidemiology of Respiratory and Allergic Disease Department (EPAR), Institute Pierre Louis of Epidemiology and Public Health, UMR-S 1136, INSERM, Paris, France; 40 UPMC, Sorbonne Universités, Medical School Saint-Antoine, 803-804-806, 8 etage/Floor 27, Rue Chaligny, CEDEX 12, 75571 Paris, France

## Abstract

The prevalence of allergic airway diseases such as asthma and rhinitis has increased dramatically to epidemic proportions worldwide. Besides air pollution from industry derived emissions and motor vehicles, the rising trend can only be explained by gross changes in the environments where we live. The world economy has been transformed over the last 25 years with developing countries being at the core of these changes. Around the planet, in both developed and developing countries, environments are undergoing profound changes. Many of these changes are considered to have negative effects on respiratory health and to enhance the frequency and severity of respiratory diseases such as asthma in the general population.

Increased concentrations of greenhouse gases, and especially carbon dioxide (CO_2_), in the atmosphere have already warmed the planet substantially, causing more severe and prolonged heat waves, variability in temperature, increased air pollution, forest fires, droughts, and floods – all of which can put the respiratory health of the public at risk. These changes in climate and air quality have a measurable impact not only on the morbidity but also the mortality of patients with asthma and other respiratory diseases. The massive increase in emissions of air pollutants due to economic and industrial growth in the last century has made air quality an environmental problem of the first order in a large number of regions of the world. A body of evidence suggests that major changes to our world are occurring and involve the atmosphere and its associated climate. These changes, including global warming induced by human activity, have an impact on the biosphere, biodiversity, and the human environment. Mitigating this huge health impact and reversing the effects of these changes are major challenges.

This statement of the World Allergy Organization (WAO) raises the importance of this health hazard and highlights the facts on climate-related health impacts, including: deaths and acute morbidity due to heat waves and extreme meteorological events; increased frequency of acute cardio-respiratory events due to higher concentrations of ground level ozone; changes in the frequency of respiratory diseases due to trans-boundary particle pollution; altered spatial and temporal distribution of allergens (pollens, molds, and mites); and some infectious disease vectors. According to this report, these impacts will not only affect those with current asthma but also increase the incidence and prevalence of allergic respiratory conditions and of asthma. The effects of climate change on respiratory allergy are still not well defined, and more studies addressing this topic are needed. Global warming is expected to affect the start, duration, and intensity of the pollen season on the one hand, and the rate of asthma exacerbations due to air pollution, respiratory infections, and/or cold air inhalation, and other conditions on the other hand.

## Introduction

There is a fairly wide body of evidence to show that the temperature of the planet Earth is increasing, as confirmed by warming of the oceans, rising sea levels, melting of glaciers, shrinking sea ice in both Polar Regions, and diminished snow cover in the Northern Hemisphere during the winter months. Most of the observed increase in global average temperatures since the mid-twentieth century is very likely due to the observed increase in anthropogenic greenhouse gas concentrations. Moreover, changes are also occurring in the amount, intensity, frequency, and type of precipitation as well as the increase of extreme weather events such as heat waves, droughts, thunderstorms, floods, and hurricanes.

The major changes to our world involve the atmosphere and its associated climate, including global warming induced by human activity, and are causing an impact on the biosphere, biodiversity, and the human environment. Observational evidence indicates that recent regional changes in climate, particularly increases in temperature, have already affected a diverse set of physical and biological systems in many parts of the world. A rapid increase has been observed in the number of hot days and severe meteorological events witnessed across the globe. Sea levels have also started to rise as an effect of a regression of the polar ice packs coupled with a decrease in pH due to the effects of increasing carbon dioxide (CO_2_) on our oceans. These events have led to water deprivation in certain areas, often associated with water degradation, which potentially could result in population migration and the effects on health that result from mass population movement.

Climate affects local and national food supplies, air and water quality, weather, economics, and many other critical health determinants. Thus, climate change represents a massive threat to global health that could affect many disease factors in the twenty-first century. Among others, climate change influences the development of asthma and allergic respiratory diseases and influences pollen and mold productions that induce allergic manifestations. There is also a link between climate change and air pollution; an individual's response to air pollution depends on the source and components of the pollution as well as on climatic agents. Some air pollution-related episodes of rhinitis and asthma exacerbation are due to climatic factors that favor the accumulation of air pollutants, such as ozone, at ground level.

Migration studies provide useful information on the role of environmental factors, including climate changes, in the development of atopy and asthma. Migrants need to be aware of the potential for developing allergies and/or asthma with the change in habitat to an area of increased prevalence of allergies [1]. Such migrations are not limited to human migration but also of plant migrations to regions that previously did not have such plants in their environments [2]. Strategies for primary prevention in high risk atopic individuals and secondary prevention guidelines should be developed for populations in developing countries as well as immigrants from such countries who move to atopy-prevalent developed countries. In recent years such guidelines have been initiated by the World Allergy Organization such as the GLAD-P [3].

Global warming affects the onset, duration, and intensity of the pollen season as well as the allergenicity of the pollen. Studies on plant responses to elevated atmospheric levels of CO_2_ indicate that plants exhibit enhanced photosynthesis and reproductive effects and produce more pollen. Moreover, the plants flower earlier in urban areas than in corresponding rural areas with earlier pollination of about 2–4 days. The key determinants of greenhouse gas emissions are energy production, transportation, agriculture, and food production and waste management; attempts at mitigating climate change will need to address each of these.

Current projections suggest that the world population will rise to 9 billion by 2050. Regarding urbanization, there are 20 cities expected to be populated by more than 10 million inhabitants by the year 2015, and two thirds of mankind are expected to live in a megalopolis by 2020. Moreover, in the last 50 years 50 % of pluvial forests on the planet have been destroyed, and each year 13 million hectares of forest are being destroyed or deteriorated. Food cultivation on wasted areas of tropical pluvial forests determined about 35 % of deforestation in countries in South America, 70 % in Africa, and 50 % in Asia. While there is some uncertainty about predicting future meteorological trends, and whatever interventions may be put in place to ameliorate climate change, it is still likely that the world will experience more hot days, fewer frost days, and more periods of heavy rain and consequent flooding. Paradoxically, it is likely that there will be more periods of drought. A huge increase in CO_2_ concentrations during the last two decades has been experienced; it is important to consider that after CO_2_ emissions are reduced and atmospheric concentrations stabilize, surface air temperature continues to rise slowly for a century or more.

The outdoor environment is complex with interaction between pollutants, both natural and manmade, and weather. Although diesel particles and combustion products are not directly influenced by climate change, a discussion is included in this review because of this interaction. The indoor environment is also directly influenced by the outdoor environment. Due to this relationship, the effects of indoor humidity and dampness are included in this review. Sources of fungi are difficult to pinpoint with some fungal species found both indoors and outdoors. Furthermore, indoor fungal spores may be derived from outdoor sources. Thus, the discussion of fungal allergens in this review includes both indoor and outdoor influences.

Finally, there are discussions related to allergy and asthma in the tropics, human migration, air filtration, violent conflict and economical aspects of climate change that may not appear germane to a review of the effects of climate change on allergic respiratory disease. The complexity of climate change on human life will likely make these issues relevant. It is difficult to comment on the effects of climate change on allergic respiratory disease in the tropics as there are limited data on the prevalence of allergy and asthma in these highly populated areas. A discussion is included as it is expected that the change in weather patterns will impact the tropics. Migration of populations is likely to be impacted in multiple ways when weather and rainfall change and migration changes the environmental effects of allergy. Air filtration is related to the indoor environment but as with mold and dampness the outside weather ultimately influences indoor allergen exposure. Conflict, competition for resources and economics all indirectly effect healthcare but are particularly important for diseases dependent on the environment. Thus, all of these topics are indirectly related to climate change and allergy and asthma.

As a consequence of the increased evidence of the link between climate change and asthma and allergies, it is important to focus on the underlying factors and bring them together in a documented version, such as this global report of the World Allergy Organization (WAO). Experts from different regions of the world have written important sections in this document on the various aspects of interaction between meteorological factors, air pollution, climate change, and human health. The contributions of these experts are appreciated. Their names and section contributions are listed in Table 1.

**Table 1:** Section authors

Air Pollution Episodes
Isabella Annesi-Maesano (Section Leader), Tari Haahtela, Stephen T Holgate, Juan José Sienra-Monge, Hasan Bayram, Gennaro D’Amato
Thunderstorm-related Asthma
Gennaro D’Amato (Section Leader), Lorenzo Cecchi, Nelson Rosário, Guy Marks, Isabella Annesi-Maesano
Sandstorm
Saleh Al-Muhsen (Section Leader), Mona Al-Ahmad, Rabih Halwani, Basam Mahboub, Fatma Al-Enezi, Hasan Bayram
Climate Change and Respiratory Allergy
Gennaro D’Amato (Section Leader), Lorenzo Cecchi, Isabella Annesi-Maesano, Karl Christian Bergmann
Pollen Allergy and Meteorological Factors
Lorenzo Cecchi (Section Leader), Jeroen TM Buters, Jae-Won Oh, Ignacio Ansotegui, Carlos Nunes, Gennaro D’Amato
Molds, Rain, Humidity, Dampness
Jeroen Douwes (Section Leader), Maximiliano Gomez, Jay Portnoy
Allergy and Asthma in the Tropics
Carlos E Baena-Cagnani (Section Leader), Dennis Ledford, Ruby Pawankar, Mario Sánchez-Borges, Elopy Sibanda
Migration and Urbanization
Nelson Rosário (Section Leader), Menachem Rottem
The Role of Air Filtration as an Environmental Control Measure for Allergic Respiratory Disease
Gennaro D’Amato (Section Leader), Carolina Vitale, Maria D’Amato
Cold Weather
Todor A. Popov (Section Leader), Louis-Philippe Boulet, Paola Michelozzi
Wildfires and Asthma
Isabella Annesi-Maesano (Section Leader), Sarah Elise Finlay, Youssouf Hassani, David J Baker
Violent Conflict and Asthma
Donata Garrasi
Economical Aspects of Climate Change
Erminia Ridolo (Section Leader), Marcello Montagni

Allergic respiratory diseases and asthma are a result of environmental and immunologic interaction. Climate change is modifying allergy and asthma in both predictable and unpredictable ways. Weather influences human behavior and botanical allergen production. The result is complex with many variables. This review summarizes these variations and hopefully will aid in the response to climate change.

This document is a product of a joint initiative of the 2012–2013 WAO Committee on Climate Change and Biodiversity and the 2012–2013 WAO Committee on Asthma.

## Air pollution episodes

### Introduction

Air pollution is an alteration of the levels of quality and purity of the air due to natural or anthropogenic emissions of chemical and biological substances. In the last century, the massive increase in emissions of air pollutants due to economic and industrial growth has made air quality a major problem for many countries in industrialized and industrializing countries and an emerging problem for the rest of the world. At the present time, it is mainly the vehicular pollution that degrades the quality of air in the cities of industrialized countries, whereas industrial pollution still constitutes the largest source of air pollution in countries undergoing industrialization. However, other sources of pollution should not be underestimated. Desert sand, sea salt, wildfires, and volcanic ash are considered as natural pollution and have to be added to levels of particulates that pollute the air. The return to wood for heating observed in the last decade is another source of pollution. Although wood energy has an indisputable advantage in regard to greenhouse gas emissions, it is a source of other gaseous and particulate pollutants. Other forms of air pollution are pollens and mold spores. In addition, people are exposed to air pollution also within premises such as houses, offices, and schools, where they spend between 80 % and 90 % of their time.

Today, tens of thousands of known or suspected air pollutants, sometimes acting in synergy with each other as well as with other parameters (temperature, wind, etc.), are taken into account. However, only those factors mandated by legislation are monitored. Indoor environments are a mixture of chemical pollutants and allergens such as paints, adhesives, flooring chemicals, cleaning products, combustion products from heaters and cooking, asbestos, animal allergens, mycotoxins, and fungal allergens. Moreover, with its 3000 compounds identified to date and its 5 billion particles per cigarette, tobacco smoke is unquestionably the most formidable of air pollutants related to human activity.

We discuss in this section the links between air pollution episodes and asthma by stressing the underlying mechanisms.

### Mechanisms

#### Air pollution and inflammation

In vitro studies suggest that air pollutants modulate various inflammatory parameters at the cellular level. Ozone has strong oxidation activity and can induce the release of inflammatory mediators such as hyaluronan [4], platelet activating factor (PAF) [5], interleukin (IL)-1β, −6, −8 and tumor necrosis factor (TNF)-α [6] from lung cells. Similarly, environmental particles have been shown to induce release of TNF-α and macrophage inflammatory protein (MIP)-2 from murine lung cells [7]. On the other hand, studies of human B cells demonstrated that diesel exhaust particles (DEP) and polyaromatic hydrocarbons (PAH) derived from DEP can induce the synthesis of IgE in the presence of IL-4 and CD40 monoclonal antibodies, suggesting that this pollutant may potentiate sensitivity to common allergens [8].

Airway epithelial cells, which form the first line of innate defense against inhaled insults, play an important role in the mechanism underlying pollutant-induced effects on airways. The studies of ozone, nitrogen dioxide (NO_2_) and DEP have demonstrated that these pollutants induce permeability of human bronchial epithelial cell cultures while inhibiting ciliary beat frequency [9–13]. As a result, this may lead to a delayed clearance of allergens and irritants inhaled to airways. Furthermore, increased permeability of airways may result in increased penetration of these agents to submucosa where they can interact with residential cells such as airway smooth muscle cells and fibroblasts as well as inflammatory cells including mast cells, eosinophils, lymphocytes, and neutrophils.

Studies of airway epithelial cells also reported inflammatory effects of air pollutants [9, 10, 12, 14]. Ozone increased the release of inflammatory mediators including PAF [5], IL-8, granulocyte macrophage-colony stimulating factor (GM-CSF), TNF-α and soluble intercellular adhesion molecule (sICAM)-1 from human bronchial epithelial cells (HBEC), which were suppressed by glutathione, an antioxidant that can be naturally produced in cells [14]. Furthermore, NO_2_ enhanced release of leukotriene (LT) C_4_, GM-CSF, TNF-α, IL-8, regulated on activation normal T cell expressed and secreted (RANTES) and sICAM-1 [9, 12, 15]. Similarly, DEP induced release of IL-8, GM-CSF and sICAM-1 from primary human bronchial epithelial cells [10]. Airway epithelial cells also express HLA-DR and CD86 and can present antigen to T cells. The expression of these co-stimulatory molecules is enhanced by DEP and mite antigen in mite allergic patients [16].

Studies of asthmatic patients have suggested that their airway epithelial cells are more susceptible to deleterious effects of air pollutants. For example, although ozone and NO_2_ did not affect the permeability of bronchial epithelial cells obtained from non-asthmatic subjects, these gases increased permeability of those cultures of asthmatics [13]. Under standard culture conditions, asthmatic bronchial epithelial cells produced greater amounts of IL-8, GM-CSF, sICAM-1 and RANTES as compared to non-asthmatic cells [11]. In addition, when these cultures were exposed to ozone, NO_2_, and DEP, the release by asthmatic cells was higher than cells of non-asthmatic subjects [11, 15].

#### Air pollutants and oxidative stress

Reports indicate that the oxidative stress is increased and the oxidant-antioxidant balance is tilted toward a proinflammatory state in chronic airway diseases such as asthma and chronic obstructive pulmonary disease (COPD) [17, 18]. On the other hand, air pollutants lead to oxidative and nitrosative stress in cell systems [19, 20]. Reports also state that inhaled ozone has a powerful oxidant capacity and that it can activate stress signaling pathways in epithelial cells and resident alveolar inflammatory cells. This mechanism involves activation of the transcription factor nuclear factor (NF)-κB and its translocation to nucleus where it binds to DNA consensus sequences in the promoters of pro-inflammatory genes that code for inflammatory cytokines and chemokines, which attract neutrophils and adhesion molecules [20]. Consequently, these molecules increase inflammatory cell recruitment into the airways and lung parenchyma and activate them for mediator secretion and the capacity to cause tissue damage.

However, there may be differences between individuals with respect to their response to inhaled ozone because of genetic differences [19, 21]. The candidate genes, as reported, included TNFα, manganese superoxide dismutase, glutathione peroxidase, NAD(P) quinone oxidoreductase, and glutathione S transferases (GST) [19]. Although data are not consistent, polymorphisms of Glutathione S-transferase Mu 1 (GSTM1), Glutathione S-transferase P 1 (GSTP1), and TNF-α reportedly have associations with asthma and air pollution [22]. Together, these findings emphasize the importance of locally available antioxidants (uric acid, albumin, reduced glutathione, vitamin C, and vitamin E) present in the lung lining fluid and epithelial barrier, as well as the protective effect that diets supplemented with antioxidants possibly provide [19]. Hence, studies have demonstrated that glutathione, an antioxidant, can be produced intracellular and can prevent ozone-induced inflammatory mediator release from human bronchial epithelial cells [9]. In vitro studies suggest that NO_2_, although less potent than ozone, can also activate oxidant pathways [16, 19].

Inhaled particles in demonstrations also induce a strong pro-inflammatory response in lung cells [10, 11, 23]. Particles activate oxidant pathways by inducing reactive oxygen species both directly and through uptake into epithelial cells and macrophages [24–26]. Recent studies reported that DEP induced nitrosative stress in epithelial cells and resident lung phagocytes through interactions of nitric oxide and superoxide anion [20]. Consequently, this leads to activation of transcription factors NF-κB and activator protein (AP)-1 that can result in increased release of inflammatory mediators [19, 26, 27]. In the short term, there is acute tissue damage with activation of the epidermal-growth-factor receptor (EGFR) pathway and evidence for organ-repair responses [28]. With the ongoing cycles of damage and repair, epithelial mucus metaplasia may occur, while ongoing cytokine and chemokine secretion contributes to airway inflammation [19]. Furthermore, recent studies reported that particles induced release of pro-inflammatory cytokines such as IL-1β and IL-8 from HBEC by increasing intracellular Ca^++^ levels [29].

#### Cell cycle and death

Studies have suggested that air pollutants cause cell toxicity and modify the cell death and cell cycle of lung cells. Ozone induced the death of fibroblasts obtained from lung biopsies of *normal* subjects [4], while attenuating viability of both macrophage-like cells [5], and alveolar macrophages harvested from bronchoalveolar lavage fluid of *normal* individuals [30]. However, studies showed that ozone also enhances proliferation of lung cells of rats by inducing cell cycle [31]. Furthermore, studies of diesel exhaust particles (DEP) and fine particulate matter (PM_2.5_) reported that *lower* doses of these pollutants induced proliferation of macrophages of mice, whereas *higher* concentrations enhanced cell death [32, 33]. Organic chemicals extracted from DEP induced oxidative stress in normal and transformed bronchial epithelial cells that led to increased expression of heme oxygenase 1, activation of the c-Jun *N*-terminal kinase cascade, IL-8 production, and induction of cytotoxicity [34]. Recent studies have demonstrated that DEP, under serum free condition, induce cell proliferation and decrease apoptosis of alveolar epithelial cells by mechanisms involving oxidative stress, inhibition of p21^CIP1/WAF1^ expression and stimulation of JNK and NF-κB pathways [27]. However, when fetal calf serum is added, effects of DEP on cell death, cell cycle, apoptosis regulating proteins, and IL-8 release were modulated through activation oxidant stress pathways, JNK and NF-κB. These findings suggest that extravasation of serum, as occurs in the inflamed airways of patients with chronic airway diseases such as asthma and COPD, may render airway epithelial cells more susceptible to the deleterious effects of air pollutants [35]. Studies of primary bronchial epithelial cells cultured from subjects with COPD supported these findings, and although DEP decreased the viability of COPD cells after 24 hours, under observation these effects were present in cells derived from non-COPD subjects after 72 hours [36].

### Asthma and air pollution

Degradation of air quality caused by one or more variable pollutants has been related to asthma. Outdoor air pollution exacerbates asthma in those who already have the condition [38]. Outdoor levels of air pollutants have been associated with asthma incidence but not clearly with asthma prevalence at the population level. In addition, a growing number of studies also show that children living in environments near traffic have increased risks of asthma symptoms, asthma exacerbations, school absences, and asthma-related hospitalizations as well as new-onset asthma [37, 38]. Recently, the Aphekom project (www.aphekom.org), by considering individual home addresses, calculated the number of cases of childhood asthma caused by living near a road with elevated traffic-related pollution and acute asthma events related to urban air pollution levels in 10 European cities [39]. Exposure to roads with high vehicular traffic, a proxy for near road traffic-related pollution, accounted for 14 % of all asthma cases in the study. Under the hypothesis of a causal relationship between near road traffic-related pollution and asthma, 15 % of all episodes of asthma symptoms were attributable to air pollution. However, without this assumption, only 2 % of asthma symptoms were attributable to air pollution. Pollutants along busy roads are responsible for a large and preventable share of chronic disease and related acute exacerbations in European urban areas.

Indoor levels of air pollutants other than environmental tobacco smoking have also been related to asthma prevalence or symptoms by sparse studies [40–43]. Consistent results support short-term (aggravation) and, although more rarely, long-term (prevalence augmentation) effects on asthma of poor air in indoor settings [40–42]. Environmental tobacco smoke is one of the most important risks for respiratory symptoms and diseases worldwide. The evidence is also reliable for indoor nitrogen dioxide and particulate matter which have been associated with asthma. Whereas formaldehyde and volatile organic compounds seem to be the main pollutants in indoor settings, relevant papers on respiratory disease are still scarce and limited to asthma and bronchitis. Molds have been associated with an increased risk of asthma and COPD. Contradictory results have been found between endotoxins and asthma. The role of phthalates, persistent organic pollutants, and flame retardants in respiratory diseases remains to be established. However, episodes of air pollution in indoor settings have not been studied. Studies focusing on indoor air pollutants should be developed to better understand their involvement in the inception and aggravation of respiratory diseases.

Mechanistic evidence from toxicological studies together with recent information on genes that predispose towards the development of asthma suggest that the link between air pollution and asthma is biologically plausible.

### Susceptible and vulnerable groups

If the entire human population is affected by the quality of the air, there is a great variability in exposure to air pollutants as well as in individual susceptibility, so that the extent of the response to air pollution and related health effects vary among individuals. Children are susceptible to the effects of air pollution because their lungs and immune systems are developing, they are more active in environments with high levels of air pollutants, and they receive higher doses of air pollutants compared to adults because of differences in breathing rates and patterns. Asthmatic children are even more susceptible because of their inflamed and hyperreactive airways. The elderly are another segment of the population potentially at higher risk of health effects of air pollution because of normal or pathological aging [40, 42]. However, few data exist on elderly populations. Besides age, other factors that contribute individual susceptibility are sex/gender, underlying diseases, smoking and diet, physical activity, body mass index (BMI), and genetic background.

In terms of increased vulnerability due to higher exposure, the Aphekom study showed that living near the trafficked roads is the cause of 15 % of asthma in children [39]. Recent data have indicated that socio-economically disadvantaged populations are another group at increased risk of air pollution effects because of proximity of air pollution sources or reduced management [37]. The health system and the use of care may also modulate the effects of air pollution.

### Opportunities for intervention to reduce the burden of asthma

#### Decreasing air pollution

Several intervention studies have indicated that decreasing air pollution leads to a reduction of the burden of asthma. Already in 1999 and 2002, McConnell et al., had suggested that if air pollution levels were reduced to match levels in the cleanest community, then annual asthma-related emergency department visit and hospitalization rates would be predicted to decrease from 22 to 6 %, asthma-related school absences could be reduced by two thirds [44], and new cases of asthma among the most active children living in polluted communities could decrease by 75 %.

Efforts to reduce downtown traffic congestion in Atlanta, Georgia (United States of America) during the Summer Olympic Games in 1996 resulted in decreased traffic density, especially during the critical morning period [45]. This was associated with a prolonged reduction in ozone pollution and significantly lower rates of childhood asthma events. Actually, peak weekday morning traffic counts dropped 22.5 % (*P* < 0.001), and the number of asthma acute care events decreased by 41.6 % (4.23 vs 2.47 daily events) in the Georgia Medicaid claims file as well as 44.1 % (1.36 vs. 0.76 daily events) in a health maintenance organization database, 11.1 % (4.77 vs. 4.24 daily events) in 2 pediatric emergency departments, and 19.1 % (2.04 vs 1.65 daily hospitalizations) in the Georgia Hospital Discharge Database. Traffic counts were significantly correlated with that day's peak ozone concentration (average *r* = 0.36 for all 4 roads examined). In multivariate regression analysis, only the reduction in asthma events recorded in the Medicaid database was significant (relative risk, 0.48; 95 % confidence interval, 0.44–0.86).

The alternative transportation strategy implemented during the 2008 Summer Olympic Games in Beijing (China) provided an opportunity to study the impact of the control measures and weather conditions on air quality and asthma morbidity. The average numbers of outpatient visits for asthma were 12.5 per day at baseline and 7.3 per day during the Olympic events [46]. Compared with baseline, the Olympic Games were associated with a significant reduction in asthma visits (RR 0.54, 95 % CI: 0.39–0.75) [47]. Other examples of asthma episodes following air pollution reduction have been observed. Taken together, these data provide support for efforts to reduce air pollution and improve health via reductions in motor vehicle traffic.

#### Consideration of antioxidant supplementation in the diet

Other intervention approaches regarding dietary supplementation with antioxidants have been explored for the possible reduction of asthma incidence or morbidity since pulmonary and systemic oxidative stress increase inflammatory responses relevant to asthma and allergy. A few small clinical trials suggest that specific antioxidants from diet or vitamin supplements might improve asthma control or lung function in asthmatic children or adults. Although meta-analyses of observational epidemiologic studies suggest associations between low dietary intake of antioxidants and higher asthma/allergy prevalence, there have been no longitudinal studies of dietary or vitamin antioxidant use by mother or child and the development of asthma or allergy. More studies focusing on supplementation of antioxidant sources are needed to evaluate whether antioxidants reduce asthma incidence or improve asthma control and to assess the possible benefits and risks of trials in vulnerable populations with known deficiencies in dietary antioxidants who have limited access to them and also are exposed to high levels of environmental sources of oxidants [48].

### Conclusions

Air pollutants exert their detrimental effects on airways and lungs by: (1) attenuating ciliary activity of airway epithelial cells, (2) increasing permeability of airway epithelium, (3) leading to inflammatory changes in cells of airways and lung parenchyma, (4) and modulating cell cycle and death of cells of respiratory system. Air pollutants show these effects by causing direct cellular injury or by inducing intracellular signaling pathways and transcription factors that are known to be sensitive to the oxidative stress. Reduction in exposures to air pollution in order to prevent asthma episodes can be approached at a policy level through changes in indoor and outdoor air pollution. Beyond clinical and public health approaches to reduce exposure, another strategy to be used before clean air goals are met is to decrease, when possible, the susceptibility of individuals to air pollution or to protect susceptible individuals.

Inadequate antioxidant defenses attributable to low levels of antioxidants (for example, vitamin C and vitamin E) or variations in the expression or function of enzymatic antioxidants (glutathione-*S*-transferases [GSTs]), offer promising chemoprevention targets to reduce the burden of asthma [49]. Emerging research indicates that dietary supplementation for individuals with low antioxidant levels is one promising approach to reducing susceptibility to air pollution. A second approach involves induction of enzymatic antioxidant defenses, especially for individuals with at-risk genetic variants of key antioxidant enzymes. Policies, prevention, advice and treatment need to protect all people and in particular the most susceptible ones. More in general, the search for mechanisms responsible for the effects of air pollution needs to be pursued in future work, so that effective prevention strategies can be implemented to protect people, especially the most vulnerable.

## Thunderstorm-related asthma

### Introduction

There is evidence that, during pollen season, thunderstorms can be associated with allergic asthma outbreaks in patients suffering from pollen allergy [50–66]. Pollen grains can be carried by thunderstorms at ground level, where, after rupture by osmotic shock, they may release into the atmosphere part of their content, including inhalable allergen-carrying cytoplasmic starch granules (<5 μ) or other paucimicronic components. Such small particles may be inhaled into the distal airways and induce asthma in subjects with pollen allergy [50, 51]. Demonstrations have shown that changes in the weather such as rain or humidity may induce hydration of pollen grains and sometimes also their fragmentation which generates atmospheric biological aerosols carrying allergens [57, 60, 64].

During the first phase of a thunderstorm, patients suffering from pollen allergy may inhale a high concentration of the allergenic material, like biological antigenic aerosols dispersed in atmosphere, which can induce (severe) asthmatic reactions in some cases [51, 55, 61–70]. Thunderstorms can induce attacks of severe asthma [65–67] and, in some circumstances, are a common cause of epidemics of exacerbations of asthma requiring attendance at the Emergency Department [64]. A study in New South Wales, Australia found that thunderstorm outflows were detected in 33 % of epidemics, compared with 3 % of non-epidemic days [64]. Furthermore, 48 % of epidemic days during late spring and summer were associated with thunderstorm outflow (development of a system). Yet, the mechanisms involved in the release of allergens from pollens during thunderstorms and associated risk should be known by physicians, not only allergists but also general physicians (GPs) and pollen allergy patients, to help with prevention. Information about the risk of an asthma attack is relevant also in subjects affected only by seasonal allergic rhinitis. In addition, there is a potential risk of thunderstorm-related relapse of asthma attacks in some patients. The increase in frequency of thunderstorms in some geographical areas due to climate change will potentially heighten the importance of thunderstorm-related asthma exacerbations [65–69].

### Allergenic pollen and pollen allergy

Though representing only a small proportion of the airborne particles present in the atmosphere, pollen grains can be causative agents of allergic respiratory responses in pollen allergic subjects, and pollen allergy due to its elevated prevalence and associated costs is now a public health problem [68–70]. In the European Union countries between 8 and 35 % of young adults show IgE serum antibodies to the most commonly encountered grass pollen allergens [71], and the cost of pollen allergy in terms of impaired work fitness, sick leave, consulting physicians, and drugs is very high.

During natural pollination, mature pollen grains are dehydrated when they are released by anthers at the dispersal time. Once the pollen grains come into contact with a wet surface, they absorb water undergoing rapid metabolic change, and the pollen allergens are rapidly released when the pollen grains come into contact with the oral, nasal, or conjunctival mucosa, thereby inducing the appearance of pollinosis symptoms in sensitized patients [72, 73]. The cytoplasmic allergens can be released into the atmosphere when the pollen bursts under osmotic shock and can create an inhalable allergenic aerosol. In particular, it has been observed [72] that fresh birch pollen can rupture in high humidity conditions and release an aerosol characterized by fragments of pollen cytoplasm ranging in microdroplets. Taylor et al., [73] observed that about 65 % of pollen grains grew a pollen tube up to 300 μm long prior to rupture and release of their cytopasmic content in the high humidity context. The released particles, such as fragmented pollen cytoplasm, form an ultra-fine aerosol. The same authors observed that grass anthers should be a site of pollen rupture and a source of fine particulate aerosols that contain pollen allergens [73].

The concentration of allergenic pollen influences the degree of symptoms, but the relationship between allergen exposure, inflammation of airways and clinical symptoms is complex, and factors other than allergens are involved [74]. Pollen grains penetrate into the upper respiratory tract but, because of their size, which is always greater than 10 μm of diameter, the grains rarely reach the lower respiratory tract. However, asthma due to pollen exposure is not infrequent.

Rainfall is usually known to remove pollen from the air but that is not always the case, because studies have revealed that allergens leave the pollen surface almost instantly, usually within seconds, upon contact with water. The discovery of airborne allergen carrying particles much smaller than pollen grains, such as those released by pollens during weather perturbations, particularly thunderstorm and rainfall, provided a possible explanation [50]. Indeed, the existence of allergen carrying airborne particles much smaller than pollen grains (paucimicronic particles) can explain bronchial symptoms affecting subjects during the pollen season or a thunderstorm [50, 55–59]. Due to their size, these paucimicronic particles can penetrate deeply into the airways thus inducing asthma in sensitized atopic subjects. In addition, in the context of paucimicronic particles there are orbicules, small granules (1–5 μm) or droplets developed from anther tissues, loaded with allergens, and there is evidence that these may play a role in allergic asthma thus contributing to form a inhalable aerosol during the pollen season [75–80].

### Thunderstorms and allergic asthma epidemics in subjects with pollen allergy during pollen season

There are descriptions of thunderstorm-related asthma outbreaks in various cities such as Birmingham (United Kingdom) [52], London (United Kingdom) [56, 59], Melbourne (Australia) [53], Wagga Wagga (Australia) [63], and Naples (Italy) [65], but there are case reports in other cities [66, 67].

One of the first observations regarding thunderstorms and asthma outbreaks was provided by Packe and Ayres [52] at the East Birmingham Hospital, Birmingham on 6 and 7 July 1983. These authors describe a striking increase in the number of asthma Emergency Department visit admissions during the period of a thunderstorm. During a period of 36 h, 26 asthma cases were treated in the Emergency Department, compared with a daily average of two or three cases in the days preceding the outbreak.

Another asthma outbreak occurred in London which coincided with a heavy thunderstorm on 24 June 1994, when a large increase was observed in the number of visits for asthma at the Emergency Departments of London and the southwest of England (United Kingdom). Several of the reported patients had a prior history of seasonal rhinitis but not asthma [56, 59]. The epidemic had a sudden onset on 24 June 1994; 640 patients with asthma or other airways diseases visited Emergency Departments during 30 h from 18.00, nearly 10 times the expected number. Over half (365) the patients were aged 21 to 40 years. A history of hay fever was recorded in 403 patients; for 283 patients this was the first known attack of asthma. A history of chronic obstructive airways disease was recorded in 12 patients. In all, 104 patients were admitted (including 5 to an intensive care unit). Six hundred and four (604) patients with wheezing and shortness of breath were seen in several departments, compared with an expected number of 66.6 [56]. These results confirm an asthma epidemic, with almost 10 times the usual number of patients presenting during 30 h and an excess of 574 patients attributable to the epidemic [56, 59]. The outbreak was not restricted to the London area, although the number of patients presenting to Emergency Departments on the night of 24 June 1994 was greater in the Thames regions than in other regions in England. Moreover, not all affected patients attended hospital, and this epidemic was the largest outbreak ever recorded.

**Fig. 1:**
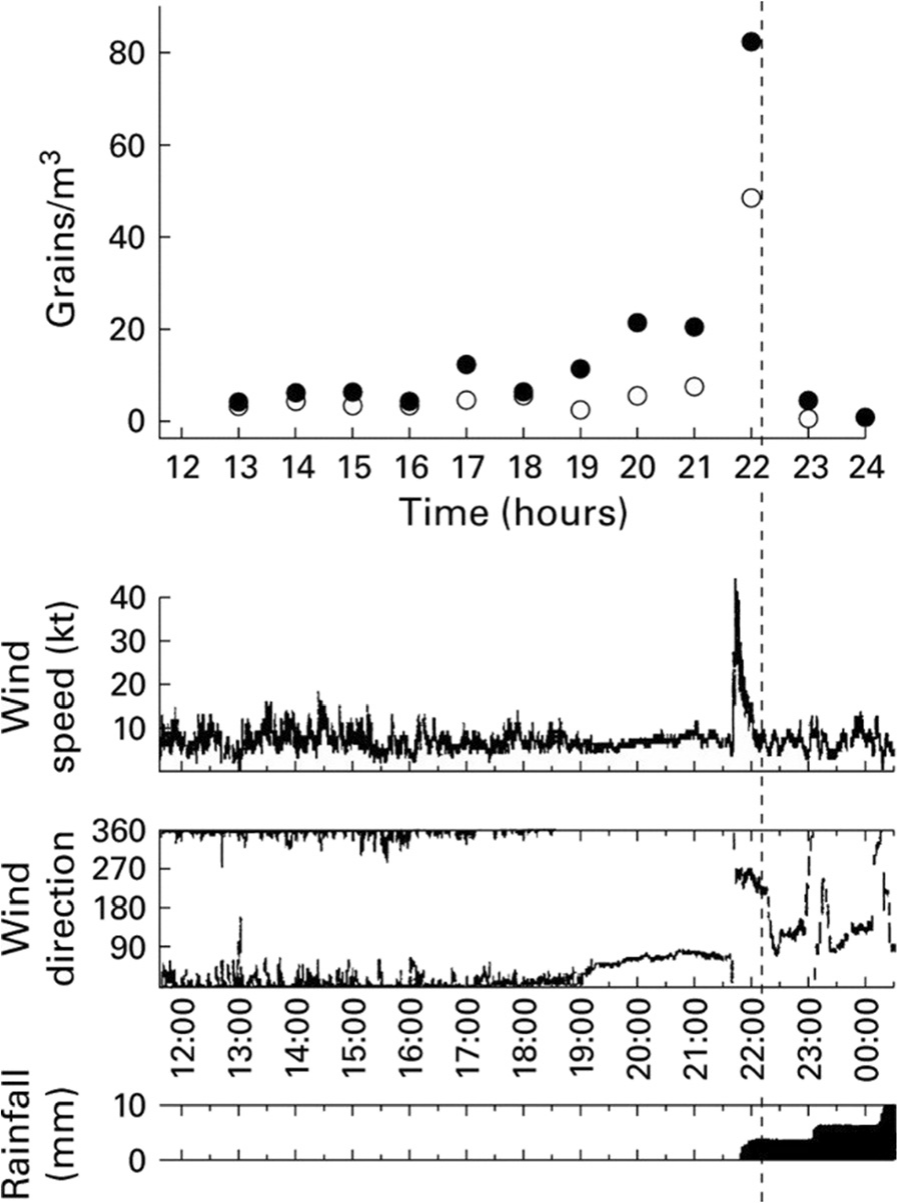
Chart showing the relation between airborne pollen concentrations and the arrival of the thunderstorm outflow in Wagga Wagga (*Australia*). Closed circles represent intact pollen grains. Open circles represent empty pollen husks. The data are plotted at the midpoint of the hour over which the concentrations have been measured. Data for wind speed, wind direction, and rainfall were recorded each minute by analogue chart recorder and have been scanned into the figure. The vertical dashed line represents the time at which the first ambulance call, seeking assistance for an attack of asthma, was received in Wagga Wagga. Examination of the recorded radar images (not shown) reveals that the line of the thunderstorms arrived at the site of the Burkard trap about 10 min before it reached the AWS. Reproduced with permission from BMJ Publishing Group Ltd [64]

Other asthma outbreaks during thunderstorms were described in Melbourne (Australia) [53], where two large asthma outbreaks coincided with thunderstorms. These events also were followed by a rapid increase in hospital or general practitioner visits for asthma. Taking into account the Melbourne experience, a similar mechanism could have been involved, although other factors may have also contributed. Further asthma outbreaks occurred in Wagga Wagga (Australia) on 30 October 1997 [63] and in Naples (Italy) on 4 June 2004 [65]. In Wagga-Wagga 215 asthmatic subjects attended the local emergency department, 41 of whom required admission to hospital. Marks et al. [64] demonstrated that the arrival of a thunderstorm outflow was accompanied by a large increase in the concentration of ruptured pollen grains in ambient air. It seems likely that the outflow of air from the upper thunderstorm cell, rather than electrical activity, thunder or rain alone, is responsible for the observed event (Fig. 1). Furthermore, 96 % of affected individuals had positive skin prick tests to rye grass pollen, compared with 64 % of other patients with asthma (adjusted odds ratio 23.0, 95 % confidence interval 6.6 to 84.3), and 90 % of thunderstorm cases reported recent hay fever symptoms compared with 69 % of other patients with asthma [63]. During the episode of thunderstorm-associated asthma registered in Naples on 4 June 2004 (between 1.30 and 2.00 am), 6 adults (3 women and 3 men between 28 and 60 years old) and a girl of 11 years had attacks of severe asthma, which in one case was nearly fatal. All patients received treatment in Emergency Departments and one was admitted to an intensive care unit for very severe bronchial obstruction and acute respiratory insufficiency. All individuals were outdoors when the thunderstorm struck. All seven patients were sensitized with allergic respiratory symptoms upon exposure to *Parietaria* pollen but were not sensitized to grasses [65]. *Parietaria* is an *Urticacea* that is widespread in the Naples area with a spring and summer pollen season, in part contemporaneous with that of grasses [68–70, 80]. During the thunderstorm, the concentration of airborne *Parietaria* pollen grains was particularly high with a peak of 144 grains/m^3^ being recorded on 3 June 2004 [65, 81].

### Possible mechanisms for thunderstorm asthma

Although much remains to be understood about the relationship between an increase in the number of asthma attacks and thunderstorms, reasonable evidence exists in favor of a causal link in patients suffering from pollen allergy [50–67, 76, 77] (Table 2). What is most frequently hypothesized is that dry updrafts entrain whole pollens into the high humidity at the cloud base of a thunderstorm where pollens may rupture and cold downdrafts carry pollen fragments to ground level. In other words, at the onset of a thunderstorm, pollen fragments are carried to ground level where outflows distribute them. As a consequence, there is a high inhalable allergen load in the air. Taylor et al., [73] hypothesized that the turbulent front of the advancing outflow releases more pollen from flowering grasses, and then may entrain them into the cloud base. Strong electric fields develop in the thunderstorm. Positive ions are released from the ground and attach to particles and electric charge may enhance pollen rupture. Grass pollens, after rupture by osmotic shock during thunderstorms, release large amounts of paucimicronic allergenic particles. Because of their very small size, these microparticles can penetrate the lower airways inducing the occurrence of bronchial allergic symptoms. Pollen grains are ruptured in rainwater by osmotic shock, with each grain releasing around 700 starch granules that are small enough to penetrate the airways and trigger asthma attacks in previously sensitized subjects. These phenomena were observed while levels of chemical air pollution were below or similar to levels in a control period.

**Table 2:** Characteristics of described epidemics of thunderstorm-associated asthma

There is a link between asthma epidemics and thunderstorms.
The epidemics related to thunderstorms are limited to seasons when there are high atmospheric concentrations of airborne allergenic pollens.
There is a close temporal association between the start of the thunderstorms and the onset of epidemics.
There are not high levels of gaseous and particulate components of air pollution during thunderstorm-related asthma outbreaks.
Subjects with pollen allergy who stay indoors with windows closed during thunderstorm are not affected.
There is a major risk for subjects who are not optimally treated for asthma; but subjects with pollen-induced allergic rhinitis and without prior asthma are also at risk.

Depending on the size of the population at risk, thunderstorm-associated asthma outbreaks may threaten the operative capacity of health services, as was the case in London and in Wagga Wagga. However, relapse of thunderstorm-related asthma attacks is possible, and there are descriptions of three different events in the same subjects in different years, as in the case of a pregnant woman which will be discussed later [81, 82]. An association between emergency visits for asthma and thunderstorms has been related to an increase in airborne fungal spore counts in Canada and the United Kingdom [83, 84]. A predictive model for hourly concentrations of atmospheric *Alternaria* and *Cladosporium* spores on days with summer storms in Poland was created through meteorological conditions and predicted spore concentration in advance on such days. Both allergic and non-allergic mechanisms have been implicated in the association between exposure to high concentrations of ruptured *Alternaria* spores and the exacerbations of asthma [86, 87]. Thunderstorm asthma does not accompany all thunderstorms. Despite published evidence being limited and highly variable in quality due to thunderstorm asthma being a rare event, there is evidence for the role of aeroallergens, meteorological features, and the impact of thunderstorm asthma on health services [87].

### Is there a risk of relapse of thunderstorm-related asthma?

Whereas there is increasing evidence of a possible link between thunderstorms and asthma, the fact that relapses of thunderstorm-related asthma are also possible is less well known. To date, the first and only reported case of relapse of near fatal thunderstorm-related asthma occurred in a 36-year old woman who was 20 weeks pregnant, affected by seasonal asthma, and sensitized to *Parietaria* pollen [82]. She had experienced near fatal asthma with a thunderstorm in June 2004 and had been treated in the emergency department of Cardarelli Hospital in Naples. She was admitted to the Emergency Department of the same hospital 7 years later, on 24 May 2011, despite appropriate treatment with oxygen and 2 g i.v. methylprednisolone on admission. Clinical parameters were similar to those during the first episode. Since the first episode the patient had avoided being outdoors during an approaching thunderstorm. The second time, an unexpected thunderstorm occurred while she was driving her motorbike, and she experienced increasing dyspnea that had to be treated in the Emergency Department some hours later. On those days, the *Parietaria* pollen count was higher than in the past 10 years in Naples during the same time of the year (260, 113, and 79.4 pollen x m^−3^ air in the previous 3 days, respectively), but no air pollutants were observed. Her symptoms became stable within a couple of weeks following a short course of treatment with oral corticosteroids [82].

### Protection against thunderstorm-induced asthma

Since a thunderstorm-induced asthma attack represents a high dose allergen challenge, and there is evidence that inhaled corticosteroids are protective against airway hyperresponsiveness, it is reasonable to hypothesize that regular treatment with inhaled corticosteroids might reduce the risk of severe thunderstorm-induced exacerbations of asthma in pollen-allergic individuals. There is no direct evidence to support this, but two studies have shown that affected patients in thunderstorm-related epidemics were unlikely to be taking inhaled corticosteroids [63, 88].

### Necessary conditions for a thunderstorm-related epidemic to occur

It has been proposed that the following four conditions are necessary for a thunderstorm-related epidemic to occur [85].

1. An abundance of grass pollen or fungi (in particular *Alternaria*)
2. A thunderstorm outflow concentrating the allergenic material at ground level near a population center
3. The formation of inhalable (<10 μm) particles either by rupture of pollen grains or germination of fungal spores
4. Exposure of sensitized individuals with untreated or uncontrolled airway hyperesponsiveness to an air mass containing the highly concentrated allergenic particles.

### Conclusions

Thunderstorm-associated asthma is a dramatic example of the allergenic potential of pollen and fungal antigens. Pollen-allergic patients who encounter the allergenic cloud would be more susceptible to undergo an asthma attack. Subjects allergic to pollen who are in the path of the thunderstorm outflow are likely to inhale airborne pollen allergens and to experience an airway asthmatic response. Relapse is also possible.

Subjects affected by pollen allergy should be informed about a possible risk of asthma attack and possible relapse at the beginning of a thunderstorm during pollen season. Subjects with a high degree of pollen allergy should consider staying indoors with closed windows if a strong thunderstorm is approaching.

## Sandstorms

### Introduction

**Fig. 2:**
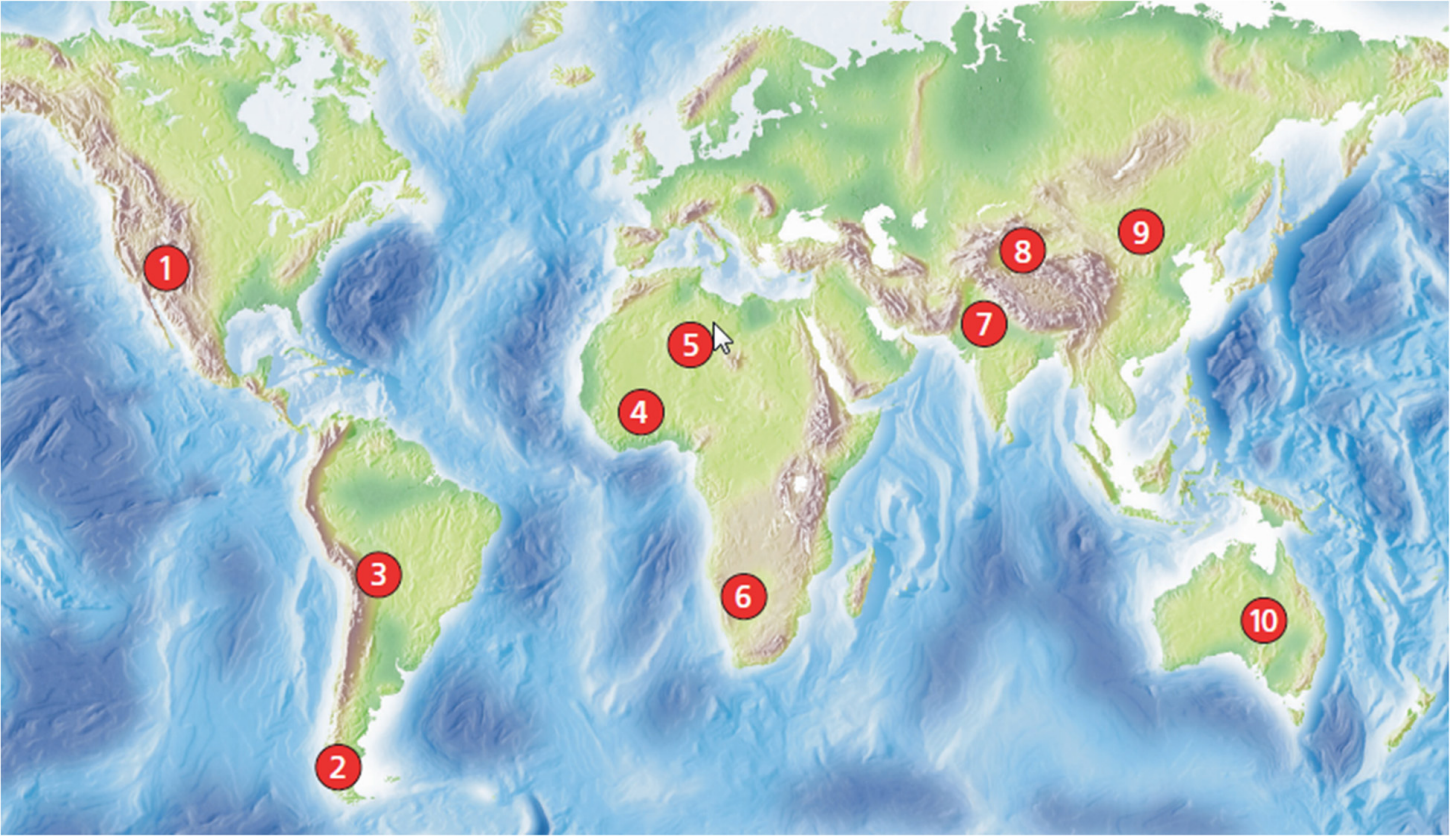
Meteorological observations and modeling have identified ten main sources of global dust events, shown in Fig. 2: (1) the Salton Sea, (2) Patagonia, (3) the Altipläno, (4) the Sahel region, (5) the Sahara Desert, (6) the Namibian desert lands, (7) the Indus Valley, (8) the Taklimakan Desert, (9) the Gobi Desert, and (10) the Lake Eyre basin. Adapted with permission from Environmental Health Perspectives [136]

A dust storm or sandstorm is a meteorological phenomenon common in arid and semi-arid regions. Dust storms arise when a gust front or other strong wind blows loose sand and dirt from a dry surface. Once airborne, smaller sand particles (<100 μm) can remain airborne for days and be transported hundreds of miles. There are nine regions that contribute to the total global production of desert dust: North Africa (Sahara), South Africa, the Arabian Peninsula, Central Asia, Western China, Eastern China, North America, South America, and Australia [89] (Fig. 2). Regions of the world in the path of dust-laden wind record increased ambient air dust concentrations that are temporally associated with deteriorations in air quality. Although estimates of the contribution of the different source areas are difficult to make, it is agreed that North Africa is the main source area with over 50 % of the total desert dust found in the atmosphere (the Sahara region accounts for 58 %), and almost five times as much as the second biggest source (the Arabian Peninsula) [89].

### Health hazards of sandstorms

Smith et al. (1999) estimated that 25–33 % of the global burden of diseases can be attributed to environmental risk factors [90] including acute respiratory infections. There is enough data on the health-related impact of the anthropogenically generated particulate matter (PM), such as PM generated by combustion engines [91]. However, not as much work has looked at the impact of naturally generated PM (such as PM emanating from dust storms).

Many studies have investigated the health risks of dust storms, and changes in morbidity, hospital admissions, or mortality before, during, and after dust events. Others compared health effects on at-risk populations compared to control groups between days with dust events and control days. A number of adverse health effects, including respiratory diseases (asthma and pneumonia), cardiovascular diseases (ischemic heart disease, cerebrovascular disease), chronic obstructive pulmonary disease (COPD), conjunctivitis, and allergic rhinitis, were shown to be associated with dust [92]. Several studies from various geographical settings also looked into the short-term health effects of dust storms with a significant association between dust storms and all-cause hospital admissions [93].

### Prevalence of asthma in regions with high sandstorm events

Asthma is a serious and prevalent public health problem that spans the entire world, with over 300 million people affected worldwide. Although Asia is only the third source area in terms of dust quantities emitted, after North Africa and the Arabian Peninsula, it is the world’s most studied region in the literature linking desert storm and air quality [94]. Several studies conducted in areas with a high rate of sandstorms, like most of the Arabian Peninsula countries, confirmed the increased prevalence of asthma in those countries. Asthma Insights and Reality in the GNE (AIRGNE) survey conducted in five Gulf Cooperation Council (GCC) countries involving 1000 patients with asthma revealed several striking findings. Use of health services in the last 12 months was high, with 23 % having been hospitalized and 52 % having attended the Emergency Department. Absence from school and work in the year was reported by 52 % of children and 30 % of adults [95]. Several other studies reported a high prevalence of asthma in these countries including Saudi Arabia (25 %) [96], UAE (15.4 %) [97], Qatar (19.8 %) [98], and Kuwait (15.6 %) [99]. The studies showed that asthma is largely uncontrolled in the Middle East, with persistent adverse effects on the psychosocial and productive life of the patient. In addition, a recent study examining more than 40,000 military personnel of the United States of America described an association between service in Iraq and the development of respiratory diseases including asthma [100].

### Sandstorm dust particles and allergen contents and their impact on the respiratory system

#### Sand microparticles

Many factors are associated with the increased effect of dust storms on the respiratory system. PM size is one of them. Samples taken in the Saudi Arabian peninsula ranged in size from less than 2 to 20 μm with more than 85 % of particles measuring less than 10 μm in diameter. Particles smaller than 2.5 μm can penetrate into the gas-exchange region of the lung. Draxler et al., (2001) reported similar findings from air samples collected at 9 sites across Kuwait and Saudi Arabia [101]. Although typically small and insoluble, these dust particles carry various soluble contaminants on their surface or within their matrix.

The histologic detection of Middle East sand particles (MESP) in rodent lungs, 6 months following a single exposure, suggests that multiple exposures may lead to a substantial lung burden and high levels of biological and metal contaminants [102]. During a single dust storm, which has an average duration of 10 h, an unprotected individual might inhale as much as 5.4 mg of dust [103]. Although coarse particles have been described to be less toxic than fine particles, they had stronger effect in inducing inflammatory mediators, which are essential in asthma attacks or other respiratory disorders [104].

#### Gaseous and mineral contaminants

Air quality is usually based on various indicators such as the mineral concentrations of SO2, NO2, O3, and CO, in total suspended particulate (TSP), and PM [103]. Depending on their origins, these particles vary in their composition (Table 3). Sahara dust, for example, is mainly made up of clay minerals, quartz, calcium, and magnesium carbonate, while particles of anthropogenic origin seem to be mainly composed of carbon-containing particles, sulphates, and nitrates. However, dust storm from the Gold Coast in Australia showed a large fraction of fine particles with an increased amount of total suspended solids, aluminum, iron, and manganese, which are common soil minerals in Australia [106] (Table 3).

**Table 3:** Origin of desert dust storms and composition of minerals (in the nine potential dust source regions in the world)

Dust source regions	Origin of dust storm	Composition of minerals
Australian dust	New South Wales and north-east South Australia	Aluminum, iron, manganese [106].
North Africa (Sahara) dust	Bodélé Depression and area covering eastern Mauritania, western Mali and southern Algeria.	Clay minerals, quartz, calcium, magnesium, carbonate [105].
Asian dust (Central Asia)	Taklamakan Desert in XinJiang Province of China and the Gobi Desert located in Inner Mongolia	Aluminosilicate, cadmium, calcium, manganese, nickel, arsenic, silicon dioxide, calcium carbonate, and organic compounds coated by nitrate [131].
Arabian Peninsula	Sudan, Iraq, Saudi Arabia and the Arabian Gulf	Mixture of silicate minerals, clays, quartz, carbonates, oxides, sulfates, and salts in varying proportions [103].
Western China	The western desert in China	Aluminum, iron, magnesium, and scandium [132]
Eastern China	Source regions to the northeast of the Tibetan Plateau	Magnesium, aluminum, silicon, potassium, calcium and iron; Illite is the principal clay mineral, and quartz [132].
North America	Central US, deserts of Arizona, cities of Yuma and Phoenix, New Mexico, Texas, and Canada during the Dust Bowl	Sulfur dioxide, nitrogen dioxide, cobalt, PM10 > 1000 μg m^␣3^ [133].
South America	Argentine loess, Southern Ocean sediments and Antarctic dust, and Puna-Altiplano plateau	Sandy silts [134].
South Africa	Namibian desert lands, Kalahari, and Karoo	Fine grained alluvial and lacustrine sediments [135].

Sand in the Middle East is primarily a mixture of silicate minerals, carbonates, oxides, sulfates, and salts in varying proportions [103]. Soil samples analyzed by Englebrecht et al., (2009) exhibited high percentages of quartz in all areas within the Middle East, except for those collected in Iraq [103]. Quartz, an amorphous and crystalline silica, is known to cause respiratory disease in occupationally exposed people and highly exposed people who live close to deserts [107]. Therefore, it may be postulated that the high concentrations of this mineral in suspended dust may give rise to allergic reactions within atopic asthmatics. Aluminum, iron and magnesium were also found to be particularly prevalent in dust samples gathered in Kuwait. Magnesium and aluminum are irritants of the mucus membranes in the proximal airways [108].

### Microbial and other allergens components

More than 200 culturable prokaryotes were detected in desert soils across the Middle East including diphtheroids, beta hemolytic bacilli, and *Bacillus* species. Lyles et al., identified over 147 bacterial isolates and 27 fungal isolates from Kuwaiti and Iraqi dust [109].

Sandstorm dust collected in Riyadh was found to contain 869 cfu (colony forming units) of fungi and 1892 cfu of bacteria per gram of dust. Comparison of these results to the colony counts in regular airborne dust indicated that, during sandstorms, fungal counts increase by 40 %, while bacterial counts increase by 100 %. The most frequently isolated bacteria were species of *Actinomyces*, *Bacillus*, *Pseudomonas*, and some coagulase-negative *Staphylococci*. Most common fungal isolates included species belonging to *Aspergillus*, *Cladosporium*, *Mucor*, *Penicillum*, *Ulocladium*, and *Alternaria* [110].

Furthermore, viruses and fungal spores are common components of Middle East dust. There are more recent demonstrations that influenza A virus can stay in ambient air during dust storms and can be long-range transported [110]. Moreover, in vitro cell culture studies suggested that sand dust enhances virus-induced cytokine secretion and viral replication in human nasal epithelial cells [111].

Date-palm pollen was also singled out as an important aeroallergen within sandstorm dust. Once inhaled, the in vivo effects of these dust particles and the biological, metal, and other regional contaminants adhering to the particle surface, are not fully understood and require further investigations [112].

### Clinical impact of sandstorm dust exposure on asthma

Dust storm events were associated with asthma and respiratory admissions to hospitals in many studies. However, the effect varies among published studies. Although many studies have shown a significant increase in asthma exacerbations and hospital admissions, others have shown only mild association with no statistical significance between asthma outcomes and dust storm exposure.

On the other hand, other studies showed that the influences of dust storms on lower respiratory tract symptoms, upper respiratory tract symptoms, ocular symptoms, and skin symptoms were often different for each dust day in patients with asthma, with only 4 % of the patients consistently showing exacerbations of lower respiratory tract symptoms on every sand storm day, and 48 % of the patients were completely unaffected by the event [113]. Factors like the levels of anthropogenic metals (such as cadmium, manganese, and nickel) and sulfur dioxide (SO_2_) can induce airway inflammation and/or bronchoconstriction [114], and these may enhance the dust storm impact on the lungs. Dust microbial contaminants can cause severe asthmatic attacks and are capable of provoking respiratory arrest in children and young adult asthmatics [115]. This is of utmost importance due to the high rates of childhood asthma in the Middle East. Similarly, pollens have been attributed to worsen asthma exacerbations during seasonal dust storms [116]. Watanabe et al., showed worsening asthma symptoms during dust dispersion period, defined as the dust storm day plus the next 6 days in April in Japan, when Japanese cedar pollen levels were increased, which was more common in patients with allergic rhinitis or atopy than in those without [116].

On the other hand, Kwaasi et al., (1998) analyzed IgE reactivity in relation to gathered sandstorm dustfall in Riyadh among three groups: normal subjects, atopic patients, and gardeners. Reactivity was particularly high among the pre-exposed gardeners (73 %) and the atopic group (68.8 %). Furthermore, skin-prick test (SPT) on the 45 atopic patients with ordinary Riyadh dust extract gave positive results in 25 % of atopic individuals, while SPT with sandstorm dust extract provoked a positive response in 47 % of these patients. This further supports the justification of pre-emptive steroid inhaler use prior to predicted sandstorms or on initial identification of the presence of a sandstorm [117].

### Clinical impact of sandstorm dust exposure on asthma in children

Among the different age groups, children are particularly sensitive to airborne exposure; however, few studies have focused on children’s health from the perspective of dust storm events [118]. Parents of schoolchildren aged between 6–14 years in Al-Ain (Abu Dhabi) were interviewed regarding the nature of their child’s asthma. They identified dust storms as one of the major precipitating factors for asthma attacks among their children. Kantani et al., reported a greater increase in hospitalizations of children than adults in Japan during dust storms, and, reflective of the Al-Ain results, recorded more admission for boys aged 6–12 years in the acute period. However, girls and infants of both sexes were more frequently admitted several days after the dust event [119]. Thalib and Al-Taiar (2012) examined the correlation between dust storms and hospital admissions due to asthma across government hospitals in Kuwait over five years [94]. They found that the only statistically significant group was that of the children 0–14 years old compared to adults. Yu et al., analyzed the effect of 76 dust storm events (Asian dust storm) during the period of 1997–2007 on respiratory clinic visits of preschool and school children. Children’s respiratory clinic visits happened 5 of the 7 days after a sandstorm event [118]. Apparently the pediatric population is the most vulnerable age group to sandstorm dust events exposure.

### Effect of sandstorm dust exposure on lung function and pathology

Numerous environmental factors have been implicated in the development of asthma, including air pollutants, tobacco smoke, aeroallergens and dust particles. During sandstorms, epithelial exposure to dust microparticles and its allergen components may result in epithelial deformity, detachment, or destruction of this vital airway barrier [120]. This may trigger signals to the underlying mesenchyme to propagate and amplify inflammatory and remodeling responses in the submucosa. In addition to the airway epithelium, airway smooth muscle responds to environmental factors, including dust and allergens via mechanisms that are independent of the immune system [121].

Murine studies have also confirmed the pathogenicity of dust particles on lung pathology. Wilfong et al., (2011) administered intratracheal dust samples collected from across Kuwait to experimental rats. Subsequent effects on the respiratory system were analyzed. Levels of total protein, neutrophils, and several cytokines and chemokines were noted to be elevated following exposure to dust particles. This data suggested a transient inflammatory response lasting several days, further strengthening the agreement for use of inhaled corticosteroids in the acute phase as well as over the following days [98]. *Ichinose* et al., (2008) found that upon removal of Asian Sand Dust (ASD) contaminants, such as sulfates (SO_4_) and nitrates (NO_3_), and microbiological materials including lipopolysaccharide and β-glucan, the resulting inflammation in murine lungs was significantly reduced, including allergen-related eosinophil recruitment [122].

### Impact of sandstorm on the development of other diseases

In addition to association with asthma, several studies indicated a clear association between dust storms and cardiovascular, respiratory dysfunction, and allergic rhinitis [123]. One of the earliest studies by Korényi-Both et al., reported an acute desert-related disease, which was manifested with hyperergic lung condition due to inhalation of fine Saudi sand dust contaminated with pigeon droppings. It was described as *desert storm pneumonitis*, and named as *Al Eskan disease* [124]. Inhabitants of deserts may develop Desert Lung Syndrome, a benign, nonprogressive fibrosis resulting from silica-containing dust depositing in the lungs following heavy exposure [125]. A correlation between metal-containing particulates and increased lower-respiratory-tract infections and chronic fibrotic lung disease has also been well documented in occupational environments [126]. Moreover, upon examining lung biopsies of 49 military personnel returning from Iraq, a more recent study suggested their development of constrictive bronchiolitis due to exposure to Iraqi dusty environments [127].

### Preventive measures and treatment approaches to asthma upon exposure to sandstorm dust

Prevention and mitigation strategies are critically important to reduce the deleterious effects of sandstorm dust on public health and vulnerable groups including asthmatic patients. Such strategies should include reducing the production of greenhouse gases (GHG), since the emission of these gases is known as the primary cause of global climate change [128], and targeting decreases in precipitation and increases in temperature and land degradation and desertification. In fact it has been demonstrated that vegetation cover can increase the surface roughness and slow or halt a dust storm [129].

A protective measure against sand dust inhalation, to some extent, could also be the use of dust masks and respirators. It seems that these masks can be used for personal protection from sand dust particles, whose size varies between 2 and 20 μM as demonstrated in samples collected from various sites in Saudi Arabia and Kuwait [101]. Air filtration systems may also be efficient in reducing air dust levels [130]. However, there is an urgent need to conduct detailed research to establish the value of such devices for public protection during sandstorm events.

Local authorities and health institutes should set up monitoring and alert systems, which could forecast sandstorms and inform communities about pending extreme weather events and dust storms. Hospitals, first step health care, day care, and emergency health care facilities should be re-organized and maintained. Physicians and healthcare personnel, as well as communities, should be educated on preventive measures and developing corrective behavior against sandstorm events. For example, asthmatic patients should be trained for specific measures to take before and during sandstorm days. Children in the Arabian Peninsula and surrounding countries are susceptible to the airborne irritants in dust storms, possibly more-so than are adults. It is hence suggested that asthmatic children’s action plans are optimized, that they always carry their reliever inhaler, and that they are familiar with its use in an appropriate manner and at an appropriate time. Physicians and public health officials should educate parents and children concerning the management of asthma during dust storms: minimizing outdoor activities, proper use of air filtration masks, and the thorough cleaning of residential areas and schools following dust storms. The community owes children the provision of a school environment that is clean and dust-free, and has proper air-flow mechanisms and insulation. However, the most important preventive measure by health professionals and physicians is with no doubt the modification of patients’ medications. In particular, the dose of anti-asthmatic drugs including inhaled steroids should be optimized before, during, and after sandstorm days.

### Conclusions

There is a growing body of evidence showing that air quality deterioration caused by desert dust storms is associated with significant impacts on human health, in particular on asthma exacerbations and respiratory hospital admissions. This effect was more pronounced on vulnerable pediatric populations with asthma. Evidence suggests that several factors related to the nature of the particulate of which these dust and sandstorms is comprised may be detrimental to the respiratory health of asthmatics. In light of the high prevalence of asthma, it is imperative that continued research is carried out into the nature of sand and dust storms, such that suitable treatment options can be defined and preventative measures may be implemented. To our knowledge, empirical evidence assessing the efficacy of medical interventions in the treatment and/or management of the effects of dust and sandstorms on asthmatics is lacking. More research is needed on this topic, particularly on the success and feasibility of medical treatment and prevention strategies.

## Climate change and respiratory allergy

### Introduction

As mentioned earlier, it is now widely accepted that the temperature of Earth is increasing, as confirmed by warming of the oceans, rising sea levels, glaciers melting, sea ice retreating in the Arctic, and diminished snow cover in the Northern Hemisphere. Moreover, changes are also occurring in the amount, intensity, frequency, and type of precipitation and in occurrence of extreme events such as heat waves, droughts, floods, and hurricanes. As stated in the recent Working Group I Report of the Intergovernmental Panel on Climate Change, “most of the observed increase in globally averaged temperatures since the mid-20th century is very likely due to the observed increase in anthropogenic greenhouse gas concentrations …” [137].

Carbon dioxide (CO_2_) is the most important anthropogenic greenhouse gas, and its atmospheric concentration has increased from a pre-industrial value of about 280 ppm to 379 ppm in 2005. About 75 % of the anthropogenic CO_2_ emissions to the atmosphere during the past 20 years resulted from fossil fuel burning; most of the rest resulted from changes in land use, especially deforestation. The same trend occurred for the other prevalent anthropogenic greenhouse gases: methane (CH_4_), and nitrous oxide (N_2_O) [137]. Major changes involving the atmosphere and the climate have a major impact on the biosphere and human environment. Many prevalent human diseases are linked to climate fluctuations, from cardiovascular mortality and respiratory illnesses due to heatwaves, to altered transmission of infectious diseases and malnutrition from crop failures [137, 138].

A number of reports on time trends in allergic respiratory diseases including asthma have shown a substantial increase in prevalence since the early 1960s. However, accumulating evidence indicates that rising trends in prevalence of asthma and atopy among adults and older children may have plateaued or even decreased after increasing for decades, especially in countries with existing high prevalence. Data from younger childhood are less reassuring [139–151].

The effects of climate changes on respiratory allergy are still unclear and current knowledge is provided by epidemiological and experimental studies on the relationship between asthma and environmental factors, like meteorological variables, airborne allergens and air pollution.

Data about the influence of weather on asthma, are poor and inconclusive. Weather affects asthma directly, acting on airways, or indirectly, influencing airborne allergens and pollutants levels. The complexity of the aerosol reaching the airways and the several compounds that play a role in this relationship might explain the controversial results of studies conducted so far.

Indoor and outdoor allergen exposure is a well-known aggravating factor for asthmatic patients even if its role in asthma development is not fully understood. Pollen grains are responsible for seasonal exacerbations of allergic asthma and rhinitis, and they disperse according to the flowering period of the plant of origin. Of great importance is the knowledge of plant geographical distribution and their flowering period and possible variations induced by climate change scenarios [77, 152–158].

Numerous studies have shown that air pollution is consistently associated with adverse health effects, and it has a quantifiable impact on respiratory diseases, cardiovascular diseases, and stroke. Data linking changes in environmental variables and changes in incidence and prevalence of asthma are still lacking, even if an increasing body of evidence shows the adverse effects of ambient air pollution on allergic respiratory diseases [139, 155–158].

The aim of this section is to briefly discuss current concepts in the field of environmental factors affecting allergic respiratory diseases and hypothesize possible long-term effects of climate change (see Table 4). Possible impacts of climate change on allergy and asthma prevalence are beyond the purposes of this work, even if the role of predictable changes in air pollution and aeroallergen exposure will be considered.

**Table 4:** Key Statements in the WAO White Book on Allergy, Update 2013, World Allergy Organization 2014, page 16. Reprinted with permission from the editors [159]

Climate Change, Migration and Allergy
The earth’s temperature is increasing as illustrated by rising sea levels, glaciers melting, warming of the oceans and diminished snow cover in the northern hemisphere.
Climate change coupled with air pollutant exposures may have potentially serious adverse consequences especially for human health in urban and polluted regions.
High summer temperatures have an impact on rates of acute exacerbation and hospital admission for elderly patients with breathing problems and may cause unexpected death.
Pollen allergy is frequently used to study the interrelationship between air pollution and respiratory allergy. Climatic factors (temperature, wind speed, humidity, thunderstorms, etc.) can affect both biological and chemical components of this interaction.
Changes in the weather such as thunderstorms during pollen seasons may induce hydration of pollen grains and their fragmentation which generates atmospheric biological aerosols carrying allergens (see also sections on thunderstorms and sandstorms in this document). As a consequence asthma outbreaks can be observed in pollinosis patients.
Migration from one country to another involves exposure to a new set of pollutants and allergens as well as changes in housing conditions, diet and accessibility to medical services which may affect migrants’ health.
Atopy and asthma are more prevalent in developed and industrialized countries compared with undeveloped and less affluent countries.
Migration studies provide information on the role of environmental factors on the development of atopy and asthma.
Physicians should be aware that environmental and climate changes may enhance the development of allergic diseases and asthma.
Physicians should be aware that migrants, especially from developing to more developed countries, are at increased risk to acquire allergic diseases and asthma and that the effect is age and time-dependent. Earlier age and longer time increase the likelihood of developing atopy and asthma.

### Weather

Data about the influence of weather on asthma are inconclusive and debated. Weather affects asthma directly, acting on airways, or indirectly, influencing airborne allergens and pollutant levels. Decrease in air temperature represents an aggravating factor of asthmatic symptoms, regardless of geo-climatic areas under study; furthermore, studies based on synoptic method (a categorization of daily weather into air mass types, which are homogeneous bodies of air with distinct thermal and moisture characteristics) supported findings derived from analyses with only air temperature as a meteorological variable. The influence of cold on airway obstruction in asthmatics is used systematically in the cold-provocation test for measuring the severity of non-specific nasal and bronchial hyperreactivity [160–162].

While results on effects of cold air on asthma are consistent, the role of humidity, wind, and rainfall is still unclear, and studies including these variables showed inconclusive and inconsistent results. Conversely, a prospective study from New Zealand has not shown any statistical significant association between meteorological variables and symptoms of asthma daily recorded in a diary [148].

### What might be the effects of climate change on respiratory allergy?

In the light of current knowledge, air pollution and aeroallergens seem critical in evaluating the possible effects of climate change on allergic respiratory diseases. An increasing body of evidence suggests that climate change might affect both environmental factors.

#### How does climate change influence air pollution type and levels?

Climate change may affect air pollutant levels in several ways: the influence on regional weather (changes of wind patterns and amount and intensity of precipitation, increase of temperature) may have an effect on severity and frequency of air pollution episodes and also on anthropogenic emissions (for example, increase of energy demand for space heating or cooling); the enhancement of the urban heat island effect may increase some secondary pollutants (i.e., ozone), and it can indirectly increase natural sources of air pollutant emissions (e.g., decomposition of vegetation, soil erosion, and wildfires) [163–168].

Tropospheric ozone (O_3_) is formed in the presence of bright sunshine and high temperatures by the reaction between volatile organic compounds (VOC) and nitrogen oxides (NOx), both emitted from natural and anthropogenic sources. An association between tropospheric ozone concentrations and temperature has been demonstrated from measurements in outdoor smog chambers and from measurements in ambient air, even if it does not occur when the ratio of VOC to NOx is low. Tropospheric ozone concentrations are increasing in most regions, and this trend is expected to continue over the next 50 years. Pollen from birch exposed to higher ozone-levels induce larger wheals and flares in skin prick test compared to lower ozone-exposed pollen suggesting an allergenicity-increasing effect of ozone [169].

Changes in temperature and precipitation may also increase frequency and severity of forest fires, sometimes with public health consequences. Changes in wind patterns may increase episodes of long distance transport of pollutants as well as of pollen grains, making large-scale circulation patterns as important as regional ones [167, 168, 170].

Climate change appears to induce an increased concentration of all health-related air pollutants. Of particular concern are potential changes in tropospheric ozone and particulate matter. However, the consequence of higher temperature on ozone concentration might be partially counterbalanced by a decrease in demand of heating systems during mild winter due to global warming. Prediction about the effects of climate change on health-related air pollution is hampered by several limits: future emissions depend on numerous factors, such as population growth, economic development, energy use and production; current knowledge about weather effects on air pollution is still unsatisfactory; there is still a need of better emission inventories and observational datasets; and long-term effects and actual enforcement of international agreements to reduce air pollution and greenhouse gases emissions (e.g., Kyoto protocol) are unpredictable.

#### How does climate change influence allergenic pollen?

Plant species require a certain amount of heat to complete their development; then air temperature plays a key role, together with other factors, such as day-length, water and nutrient availability, and soil type. Increasing worldwide evidence demonstrates that the timing of life cycle events of a large number of species have responded to the increase in Earth’s temperature. Changes involve also plants producing allergenic pollen, with expected consequences on atopic populations [70, 170–173]. Data provided by 30 years of observations within the International Phenological Gardens network showed that spring events advanced by six days, the highest rate of phenological changes observed in the Western Europe and Baltic regions [174]. Conversely, phenological trends appear to be different in the eastern border of Europe, sometimes showing 1–2 weeks later in the start of the phases. An earlier start of the season was confirmed in studies focused on allergenic plants, as birch, mugworth (*Urticaceae*, grass and Japanese cedar), even if different methods and different lengths of datasets have been used. Due to the earlier onset, the pollen seasons are more often interrupted by adverse weather conditions in late winter/early spring [70, 174–181]. Duration of the pollen season is also extended, especially in summer and in late flowering species. Moreover, there is some evidence of significantly stronger allergenicity in pollen from trees growing at increased temperatures.

Increasing CO_2_ and temperature seem to substantially increase pollen production from ragweed in experimental conditions. The same results were obtained in a study on ragweed plants growing in urban and rural areas, providing an elegant “natural” model for evaluating possible effects of global warming. In this study, *Ziska* et al., showed that Amb 1 concentration in ragweed pollen increased as a function of CO_2_ concentration. The difference in Amb 1 as a result of increased CO2 may be affected also by influences among and within different ragweed populations [175]. The observed long-term changes in the large-scale atmospheric circulation have an impact on wind patterns. These changes may increase the long distance transport of allergenic pollen into less affected areas, as shown with the detection of ragweed in central Italy [170].

However, other factors might modify the distribution of allergenic plants in Europe. The cases of grass and ragweed are explanatory. Grass pollen is responsible for a high percentage of pollinosis worldwide, and variations in total pollen count have been observed in the last decades. The analysis of the time series of aerobiological data from the United Kingdom, the longest of the world, showed a decreasing trend in terms of yearly grass pollen counts and severity of pollen season and an earlier start of the season. However there are remarkable differences between the three study sites – Cardiff, London, and Derby, underlining the role of local determinants (such as climate), plant adaptation (i.e., tolerance to environmental factors), changes in land use, and changes in species and effects of air pollutants [165].

Since the last decades of the nineteenth century, ragweed has been increasingly important from an allergological point of view, covering large areas of Central and Eastern Europe. In the eastern countries the expansion of ragweed seems to be associated with major socio-economic transitions rather than with climate change [177–181].

Recent analysis of a continental-scale pollen data set reveals an increasing trend in the yearly amount of airborne pollen for many but not all taxa in Europe, which is more pronounced in urban rather than semi-rural/rural areas. Climate change may contribute to these changes; however, increased temperatures do not appear to be a major influencing factor. Instead, we suggest the anthropogenic rise of atmospheric CO_2_ levels may be influential [182].

#### Overall effects on respiratory allergy

Several factors make a prediction very challenging in this field. Firstly, climate change scenarios are based on mathematical models, which contain some limits. Since little increases in Earth’s temperature seem to have a dramatic effect on living organisms, little differences in the magnitude of global warming might substantially change our previsions on the effects on human health. Moreover, predictions appear to be more accurate on a regional than global scale, since local factors play a key role in determining the effects of climate change.

Secondly, changes in health-related air pollutants levels are strictly dependent on future emission scenarios, which are based on economic and demographic projections. A further limit lies in the difficult-to-predict actual enforcement of international agreements to reduce emissions.

Thirdly, even if an increasing number of observations show that climate change is affecting all phases of plant development, there are still uncertainties about the effects on start, length, and end of the pollen season of allergenic plants. The numerous papers published on this topic showed the effects to be different for each plant and geographical area under study, biological and socioeconomic factors playing also a role [70, 78, 139–143]. Only a few experimental studies reported an increase in pollen allergenicity in CO_2_ enriched environment, suggesting this should be a key topic for further research over the next decade.

**Fig. 3:**
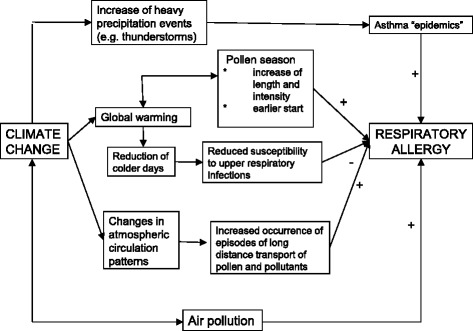
Possible effects of climate change on respiratory allergy. Source: Lorenzo Cecchi, original drawing

Finally, trends of worldwide asthma prevalence are still unclear, being different in developing and in industrialized countries. Epidemiologic studies show that air pollution has a negative effect on lung development, potentially explaining the association of pollution on the greater prevalence of asthma in urban environments [70, 159, 175–179, 182, 183]. The influence of climate change on symptoms of respiratory allergy is still unpredictable. Two opposite effects could be relevant. On one hand, global warming could increase length and severity of pollen season and, as a consequence, of pollen allergy. Moreover, the increase in occurrence of heavy precipitation events, as predicted by recent climate change scenarios, could make episodes of asthma epidemics more frequent. Finally, overall effects on health-related air pollutants seem favorable to an increase of urban air pollution episodes. On the other hand, increase of Earth’s temperature could reduce the effects of cold air on asthma and rhinitis, also making patients less susceptible to upper respiratory infections (Fig. 3).

### Conclusions

Pollen allergy is frequently used to study the interrelationship between air pollution and respiratory allergy. Climatic factors (temperature, wind speed, humidity, and thunderstorms, etc.) can affect both components (biological and chemical) of this interaction. By attaching to the surface of pollen grains and of plant-derived particles of paucimicronic size, components of air pollution could modify not only the morphology of these allergen-carrying agents but also their allergenic potential. In addition, by inducing airway inflammation, which increases airway permeability, pollutants overcome the mucosal barrier and could be able to “prime” allergen-induced responses.

Much remains to be studied by using biologic, genetic, epidemiologic and clinical approaches to air pollution. However, public health approaches to decrease exposure of citizens to air pollution must be implemented considering that the following goals should be reached:

- Reducing use of fossil fuels and controlling vehicle emissions
- Reducing the private traffic in towns improving the public transport and favoring pedestrian traffic
- Planting non-allergenic trees in cities [183].

As for the last point, it is important to consider that unfortunately each year a lot of hectares of woods are destroyed by prevalent fires, frequently criminally intentional. Of course, these fires and the reduced woods are responsible for the reduction of living trees. Moreover, although there is no general agreement, increasing the antioxidant defenses of the human airways by eating antioxidant foods should be implemented.

Governments worldwide and international organizations such as the World Health Organization and European Union are facing a growing problem of the respiratory effects induced by gaseous and particulate pollutants arising from motor vehicle emissions. The last release of the Intergovernmental Panel on Climate Change (IPCC) stated that climate change is very likely due to human activity, according to an impressive amount of data published in the last years, and policymakers seem now to take into greater consideration preventive measures (such as the Kyoto Protocol) and alternative energy sources. Desirable positive effects of these measures may be achieved in the next decades but, presumably, global temperature will continue to increase in a short-term perspective.

## Pollen allergy and meteorological factors

### Introduction

Pollen grains, a causative agent of asthma and rhinitis, are among the commonest allergens in atopic patients. Pollen allergy is now a public health problem due to its elevated prevalence and associated costs [70] in terms of impaired work fitness, sick leave, and cost of healthcare [159]. In the European Union countries between 8 and 35 % of young adults show IgE serum antibodies to grass pollen allergens that are the most commonly encountered [71]. Major pollens involved in rhinitis and asthma besides grass include birch primarily in northern countries and ragweed in east-central Europe. In southern countries *Parietaria* (wall pellitory) and cypress play an important role as well.

### Pollen and weather monitoring

Weather conditions, including rainfall, atmospheric temperature, humidity, wind speed, and wind direction, may alter the concentrations of plant pollens and other allergens, which can subsequently influence the occurrence of allergic diseases such as asthma, allergic rhinitis, allergic conjunctivitis, and even atopic dermatitis [155, 184–188]. Many studies have demonstrated that CO_2_ concentration and increased atmospheric temperature increase pollen concentration [189].

Most work on the impacts of climate change on aeroallergens can be divided into a number of distinct areas, including impacts on pollen amount, pollen allergenicity, pollen season, plants, and pollen distribution. Metrological factors such as mean temperature, wind speed, humidity, amount of sunlight, and degree-days can directly affect biological and chemical components of this interaction [190, 191].

The accumulation of daily sunshine, and other impacting meteorology factors such as humidity and precipitation, should be taken into consideration to further improve the accuracy of the modelled start dates and season lengths of birch and oak pollen and develop regression models for pollen prediction. Daily fluctuations in the amount of pollen have to do with a variety of meteorological factors such as temperature, rainfall, and the duration of sunshine. Temperature and rainfall are especially important in determining pollen concentrations, but the relationship is complex and influenced by other variables. At least 10 weather elements that are thought to affect the concentration of pollen are used to develop equations for the pollen forecasts. The elements are: daily mean temperature, rainfall, average wind speed, relative humidity, maximum temperature, minimum temperature, temperature range, continued rainfall hours, accumulated sunshine hours, and accumulated mean temperature. Predictive equations for each pollen species and month are developed based on statistical analyses using observed data with 10 weather elements during the last several years in some countries. Although few observations and estimates were reported regarding season start and length of allergic pollens in the other countries, earlier start dates and rising pollen concentrations have been reported widely in many European countries [192, 193].

The growing degree hour (GDH) model was used to establish a relationship between start and end dates of pollen production and differential temperature sums using observed hourly temperatures from surrounding meteorology stations. Studies of climate change effects on distributions of allergenic pollens have focused typically on analysis of observed airborne pollen counts and their regression relationships with local meteorological and climatic factors. Observed airborne pollen data for pollen collecting stations at locations representing a wide range of geographic and climatic conditions should be analyzed statistically to identify the trends of start date, season length, and annual mean and peak value of daily concentrations of allergic pollen [194].

### Effects of weather on pollen release/flight

Pollen season, that is the period during which pollen is present in the air, is related to the flowering season, for pollen has to be previously produced and emitted by mature flowers. Pollen seasons and flowering seasons usually do not fully coincide because of the effects of mid- and/or long-range transport. The onset, duration and intensity (i.e. abundance of pollen grains in the air) of the pollen season vary from year to year. Weather variables, mainly air temperature, sunlight and rainfall together with CO_2_ are among the main factors affecting phenology (that means the times of the appearance of first leaves, first flowers, autumn leaf coloration, and so on) and pollen production by plant. In addition, weather patterns influence the movement and dispersion of all aeroallergens in the atmosphere through the action of winds and rainfall and depending on the atmospheric stability [195].

The effect of temperature is greater on the spring and early summer flowering plants whereas species that flower in late summer and fall generally are correlated with photo period. Thus, the former species are more affected by warmer winters and springs, showing an earlier flowering in the last two decades [196].

The complex relationship between weather, climate and concentration of pollen in the atmosphere play a key role on the allergen level. In order to reduce the exposure to pollen and to improve the measures listed earlier, it is therefore crucial to provide patients with a reliable aerobiological forecast. Although the quality of weather forecast improved substantially in the last decades, effects of weather variables on aeroallergen load are not completely understood. For instance, rainfall clears the air from pollen, thus reducing the risk of exacerbation of rhinitis and/or asthma; extreme rainfall events, like thunderstorms, are, however, associated with severe/near fatal asthma epidemics [81]. A deeper understanding of the relationship between weather and pollen concentration is the first step for the development of a reliable pollen forecast, useful in preventing pollen dependent allergic symptoms.

### Effects of weather on allergen load and pollen potency

In the last 10 years allergology has changed dramatically, moving from the extracts to a molecular-based diagnosis. This dramatic change prompted a new way to look at the mechanisms linking exposure, sensitization and symptoms of respiratory allergic diseases, involving aerobiology [197]. Although the pollen count has been used for over 50 years for the assessment of allergen exposure both in clinical practice and experimental studies, proof is lacking that pollen count is representative for allergen exposure. In this context, measurement of the allergen content of pollen, the so called *pollen potency*, might contribute to improve our knowledge on the relationship between exposure to airborne allergens and allergic sensitization and symptoms. After the early studies at the end of the twentieth century [198, 199] a number of papers have been published in the last few years in Germany and Spain [200–205] utilizing improved sampling devices and methods of analysis [206].

More recently, the HIALINE (Health Impacts of airborne ALergen Information Network) (www.hialine.eu) project funded by the European Union set the standard for the assessment of pollen potency in ten countries over three pollen seasons. Measurement of Phl p 5, Ole e 1 [207] and Bet v 1 [208] in the pollen of grass, olive tree, and birch respectively confirmed that the allergen count deviates from pollen count. These findings imply that potency of pollen is variable, even on a daily basis, and that number of pollen might not best represent allergen exposure.

Both climate and weather play a key role in the production, release and bioavailability of pollen-derived allergens. The explanation why pollen vary in their potency is that the allergens are synthesized in pollen during ripening of the pollen. In the week before pollination, no allergen was found in pollen, which otherwise were ready to release. Subsequently, a rapid increase in allergen content of the pollen occurred. At the same time, the anthers developed. Anthers react to weather conditions, and when conditions are good for pollination (ripe anthers, low humidity and warm enough) they open and release pollen. If the weather conditions are favorable for early pollination, ripe anthers will release less ripe pollen with less allergen. Otherwise, when weather is such that anthers do not open until later, riper pollen with more allergen is released [201]. The meteorological factors that control allergen ripening are not yet clear, as studies performed so far compared weather condition around the pollen trap with the potency or amount of detected pollen. This assumes that the detected pollen stem from plants that live in the area for which the temperature is recorded. However, pollen travel longer distances and the weather condition at the source [209], i.e., at the place of pollination, must be correlated with pollen potency. This has not been done.

### Advice and tips for reducing exposure to pollen

Patients should recognize their allergy symptoms and if they still have not received a diagnosis should seek necessary medical help, preferably from a knowledgeable physician. It is important to be aware of both the pollens to which a patient is exposed and those to which the patient is sensitized.

The patients sensitized to pollens should read their regional pollen bulletins to keep themselves properly up-to-date on allergic disease prevention and treatment. This allows them to associate their symptoms to the pollen they are allergic to, understand the disease better and consequently treat it more efficiently. While knowledge of allergic disease is becoming more widespread, it is noted that many allergic patients are underdiagnosed and undertreated.

After diagnosis, the patient ideally should be treated prior to or at the beginning of the seasonal period to minimize the severity of the disease. Usually oral anti-histamine medication or/and nasal steroids can help and reduce symptoms, mainly on those suffering of allergic rhinitis. This should be adapted on a case-by-case basis in line with the symptoms in question and always following the physician's instructions. Certain pollen-allergy patients can benefit from taking anti-allergic vaccines, which are very effective if used appropriately and under the strict supervision of a knowledgeable physician. Taking these vaccines can change the course of the allergic disease which can provide primary prevention of more severe signs, such as asthma.

In addition to pharmacological treatment, the other element necessary for the control of an allergic disease is to avoid exposure to the allergens to which the individual is sensitized. Unfortunately, in the case of pollen allergy, an effective and total avoidance is not possible. However, patients sensitized to pollens, suffering from allergic rhinitis, rhino-conjunctivitis, or asthma, can take several measures to provide protection against or to minimize the symptoms. These are as follows:

- Know the times of the year when pollen is most likely to be a problem
- Try to stay indoors when the wind speed is high
- Reduce outdoor activity, especially in high pollination areas
- Avoid outdoor sports or camping
- Avoid walking in large grassy areas or mowing the grass
- Keep the windows closed when the pollen counts are high, particularly on hot and dry days with strong winds
- Use high-strength particle filters in cars and driving with the windows closed
- Cyclists and motards use closed-visor helmets
- Wear dark glasses outdoors
- Change clothes and take shower after extended outdoor activity during high pollen production time.

## Molds, rain, humidity, dampness

### Introduction

There is a cumulative body of evidence demonstrating a highly consistent association between home dampness and respiratory symptoms and asthma [210]. Indoor dampness may not only aggravate pre-existing respiratory conditions, but there is also (limited) evidence that it may cause new onset symptoms and asthma [211]. It may also increase the risk of respiratory infections [212] and depression [213] which in turn may increase the risk of respiratory symptoms and asthma [214, 215].

Estimates from several European countries, the United States of America, and Canada suggest that at least 20 % of buildings have one or more signs of problems with dampness [216]. These estimates are mostly based on self-reports of leaks, floods, wet basements, window condensation, visible fungal growth, or moldy odors. Based on several studies conducted in the United States of America, Mudarri and Fisk estimated the prevalence of dampness and/or fungi in US houses to be as high as 50 % [217]. A 2011 World Health Organization report estimated that in cold climates 15 % of dwellings have signs of dampness problems in general, and 5 % have signs of mold problems [218]. In warm climates the corresponding estimates were 20 % for dampness and 25 % for mold. The estimate for water damage was 10 %, regardless of climate. Since dampness is more likely to occur in houses that are overcrowded and lack appropriate heating, ventilation, and insulation [210], the prevalence of damp indoor problems in low-income communities and rental accommodation scan be substantially higher than the national average.

Thus, a substantial proportion of the population is currently at risk of experiencing adverse health effects associated with damp indoor environments. Climate change and its effects on the weather (i.e., storms and heavy rain fall) and the subsequent rise in sea levels and increased frequency and duration of floods are likely to further increase the proportion of buildings with damp problems, and exacerbate the defects in buildings that are already leaking, particularly in flood-prone areas such as river valleys and coastal areas. In addition, the high energy costs may prevent adequate heating in winter in many homes (fuel poverty) leading to increased risk of condensation and indoor dampness. Climate change through increased temperatures and humidity may also increase the atmospheric concentration of fungal spores combined with an earlier seasonal start and increased allergenicity further increasing the risk of allergies and asthma [188].

In this section, we discuss the respiratory health effects associated with damp and mold-affected indoor environments and the impact climate change may have on exposure to mold and dampness, and associated effects on asthma.

### Health effects of indoor dampness

A consistent association between home dampness and respiratory symptoms and asthma has been observed in a large number of studies conducted across many geographical regions [210, 211]. Positive associations have shown up in infants [219], children [220] and adults [221], and there are demonstrations of some evidence for dose–response relationships [210, 211, 222]. Based on a comprehensive literature review up to July 1998, Bornehag et al., concluded that indoor dampness “appears to increase the risk for health effects in the airways, such as cough, wheeze and asthma” [223]. The risk was estimated to be 1.4–2.2 fold compared to those not living in damp homes. The authors also suggested an association between dampness and other symptoms such as tiredness, headache, and airways infections. A review by the Institute of Medicine confirmed this picture and stated that there was sufficient evidence of an association between indoor dampness and upper respiratory (nasal and throat) tract symptoms, cough, wheeze, and asthma symptoms in sensitized persons [210]. However, the report noted that there was insufficient evidence to conclude that the association was causal. A more recent review including publications up to the end of 2009 confirmed this but commented that the evidence for an association with asthma exacerbation was now strongly suggestive of being causal [211]. Fisk et al., in another recent review concluded that “building dampness and mold are associated with approximately 30–50 % increases in a variety of respiratory and asthma-related health outcomes” [217]. A recent formal meta-analysis including studies published from 1980 to 2010 showed that visible mold was positively associated with asthma (OR 1.49 (95 % CI 1.28-1.72), wheeze (OR 1.68 (95 % CI 1.48-1.90) and allergic rhinitis (OR 1.39 (95 % CI 1.28-1.51) [224].

Only a few experimental intervention studies (in subjects with respiratory symptoms) have been conducted and these showed reductions in asthma exacerbations, acute care visits, self-reported poor health, wheeze, school and work absenteeism when compared to control groups [211]. Of particular interest is a study by Kercsmar et al., who conducted a controlled experimental intervention study in houses of highly symptomatic children [225]. Removal of the sources of dampness and mold caused a substantial and statistically significant reduction in asthma exacerbations. Also, acute care visits at 6–12 months after intervention were 90 % fewer in the remediation group when compared to the control group.

Although a causal association between dampness and respiratory morbidity has been suggested by some authors [223], it is based mainly on cross-sectional studies and prevalence case–control studies. Therefore it is not clear whether indoor dampness causes *only* exacerbates pre-existing respiratory conditions such as asthma. Longitudinal and incident case–control studies are better suited to assess this; however, only few of these studies have been conducted. Three longitudinal studies following babies from birth showed that indicators of home dampness were associated with an increased risk of asthma symptoms in the first 1 or 2 years of life [211]. These studies must be interpreted in light of the difficulty in diagnosing asthma in infants. Two other prospective studies in older children (1–13 years) confirmed that exposure to indoor dampness was associated with the subsequent development of asthma [211]. These studies showed that dampness increased the risk of new onset asthma by a factor of approximately 2.5. Also, a longitudinal study in 6443 adults showed that building dampness (measured either as water leakage or damp spots), but not visible mold was associated with accelerated lung function decline in women [226]. Although these results require confirmation, they do suggest that in addition to new onset asthma, dampness may also cause a decline in lung function.

In addition to non-infectious respiratory symptoms, there are also a number of studies in children and adults, suggesting an association between indoor dampness and respiratory infections [212]. These studies included two prospective studies in children showing that dampness increased the risk by a factor of 1.3–5.1 [212]. As respiratory infections particularly in early life are associated with asthma development this may further increase the risk of asthma [214]. Likewise, flooding and indoor mold and dampness are associated with adverse effects on mental health [213] which in turn may increase the risk of asthma [215].

### Indoor exposures associated with indoor dampness

The causal exposures associated with dampness and adverse health effects have not been conclusively determined, but a critical role for bioaerosols (most notably mold and bacteria) is plausible [211]. Bioaerosols are generally found in elevated concentrations in the air in damp and poorly ventilated buildings. There are a broad range of bioaerosols relevant to damp indoor environments, which include fungi (especially molds and yeasts), fungal spores, hyphae, as well as fungal fragments and allergens; bacteria and bacterial spores; microbial toxins and pro-inflammatory components, such as mycotoxins, ergosterols, β 1 → 3-D glucans and endotoxins; mite allergens; algae; amoebae; and viruses. In addition to bioaerosols, indoor dampness may result in elevated concentrations of fungal volatile organic components as well as increased chemical emissions of building materials and floor covers. The interest in bioaerosol exposure has increased in more recent times, in particular due the global increase in allergies and asthma. However, despite the recognition of the importance of bioaerosols, the precise role of these agents in the induction, aggravation and progression of asthma (and other respiratory symptoms) is only poorly understood. Although other bioaerosols may also play a role, the specific focus of this section is on molds.

### Asthma burden due to indoor dampness and mold

A 2011 World Health Organization report has shown that a considerable proportion of childhood asthma cases is attributable to exposure to indoor dampness and mold [218]. Based on data for 45 countries of the European region, it was estimated that 0.07 asthma-related deaths and 50 asthma-related disability-adjusted life years (DALYs) per 100,000 children per year are associated with exposure to dampness in dwellings, and that 0.06 deaths and 40 DALYs per 100,000 children per year are associated with exposure to mold [218]. These two environmental burden of disease assessments overlap, as damp indoor conditions are a prerequisite for microbial growth. As noted above, the burden appears to be highest in low-income communities often relying on poorly heated and maintained rental accommodation. Adults with current asthma were more likely to be black and to live in households that were <100 % federal poverty line (FPL) [226, 227].

### Atmospheric mold exposures and asthma

Outdoor exposure to fungal spores of *Alternaria* and *Cladosporium* has been associated with atopic sensitization and asthma, with the strongest evidence for asthma exacerbations. In particular, several studies in children and adults have shown that increases in atmospheric fungal spore counts were associated with significantly more asthma admissions and emergency visits [228, 229]. Similarly, positive associations have been observed for asthma symptoms, asthma medication use, and peak flow variability [229], although results have not always been consistent with some studies finding no associations [229]. There is also evidence that increased atmospheric mold spore concentrations may play a role in *thunderstorm asthma*, a phenomenon characterized by a sharp increase in asthma hospital emergency visits following thunderstorms. In particular, a Canadian study found that fungal spore counts doubled and pediatric asthma emergency admissions increased by more than 15 % on days with thunderstorms while no increases were found in the concentration of pollens and other air pollutants [83]. These studies suggest that, in addition to indoor sources of mold, atmospheric mold may also be a risk factor for asthma (exacerbations).

### The effects of climate change on dampness and indoor and ambient mold exposures

The impact of climate change on dampness and indoor and outdoor mold has received little attention in comparison to other aeroallergens such as pollens. Nonetheless, there is some evidence that climate change may increase the prevalence and severity of dampness and indoor and atmospheric mold exposures which we will briefly summarize below.

As noted above, climate change and its effects on the weather (i.e., frequent storms and heavy rainfall) and the rise in sea levels and the subsequent increased frequency and duration of floods will likely affect the number of buildings with damp and mold problems and exacerbate the defects in buildings that are already leaking. However, the magnitude of the increase of houses affected by dampness and mold and their effects on respiratory health is still unclear, in part because studies evaluating the effect on indoor mold concentrations and respiratory symptoms due to flooding or water damage following severe storms are rare. The best data available are from studies assessing mold exposure and health effects following hurricanes Katrina and Rita in the United States of America. These studies showed increased fungal growth in houses that were moderately to severely affected, and there was some suggestion that the composition of the microflora also changed after the hurricanes [230]. Subsequent studies in homes with limited flood damage and with a large proportion of homes having undergone renovation work showed low levels of indoor mold spores and no differences were found between flooded and non-flooded houses [231]. Workers involved in renovation work immediately after the hurricanes were exposed to increased levels of bacterial and fungal agents, but levels markedly decreased within the first year after the hurricanes [232]. These studies suggest that increased exposure to mold following a flooding is likely, but exposure levels may return back to normal over time (at least in part due to renovation work conducted during this time). The benefits from this are diminished by the increased likelihood that subsequent flooding is more likely to occur due to climate change. The effects on asthma are not clear with some reports suggesting an increased risk of respiratory symptoms in home occupants [233] and renovation workers [234], and others showing no association [235].

It is clear that climate and weather have an effect on atmospheric molds, both in terms of number of spores and species [236]; however, only few studies have studied the impact of climate change. A study from New England (United States of America) showed both an earlier seasonal start (by 2–4 weeks) of atmospheric mold spores and higher spore counts following an El Niño event [237]. Corden and Millington examined long-term (1970–1998) trends of *Alternaria* spore concentrations in Derby (United Kingdom) and showed that spore concentrations increased with increases in local temperature [238]. A similar association was found with earlier start and increased duration of the spore season. Interestingly, there is also experimental evidence showing that *Alternaria alternata* grown at increased CO_2_ levels produced almost three times more spores and more than twice the total antigenic protein [239]. These studies provide (limited) evidence that climate change may affect the atmospheric concentration of fungal spores and start and duration of spore seasons as well as the allergenicity of fungal spores, potentially increasing the risk of allergies and asthma.

### Adaptation measures to reduce the impact of climate change on indoor dampness and mold

Optimal prevention involves measures to reduce greenhouse gases. However, in the meantime adaptation measures are essential to minimize the effects of climate change on indoor dampness and its associated respiratory health burden. These may involve a range of legislative and specific building design and construction measures to minimize the risks of leaks and surface flooding including: modification of regulations and building codes to better protect residential and commercial buildings from severe weather events and more stringent enforcement of existing and new regulations and building codes; restriction of building construction in flood-prone areas such as low-lying coastal and river areas; creation of incentives to improve existing housing stock; improvement of control of rain and surface water through appropriate drainage including drainage of the building foundations; restriction of the use of flat roofs and installation of eaves to protect walls from getting wet from rain water; the utilization of appropriate methods to ensure that buildings are watertight; installation of appropriate ventilation including in attic and crawl spaces to reduce the effects of indoor dampness; and installation of vapor barriers in walls. The effects of inadequate heating in winter due to fuel poverty (leading to increased risk of condensation and indoor dampness and respiratory symptoms) may be partially mitigated by the creation of incentives to introduce more fuel efficient heating sources (which in turn would contribute to lower green-house gas emissions). Nonetheless, even if adaptation measures are taken then some flooding and/or rain damage will be inevitable. In those cases the effects of flooding and leaks on indoor dampness and microbial growth can be minimized by early and adequate building repairs avoiding more serious problems and reducing the risk of subsequent respiratory morbidity.

### Conclusions

There is strong evidence of an association between indoor dampness and a wide range of respiratory symptoms including asthma. In addition to exacerbation of pre-existing asthma, there is (limited) evidence that indoor dampness may cause new onset asthma. Indoor dampness and flooding may also indirectly be a risk factor for asthma through increased infections and mental health problems. Climate change and its effects on the weather (i.e., storms and heavy rainfall), the subsequent rise in sea levels and increased frequency and duration of floods, and the concurrent increase in fuel poverty will likely contribute to a higher proportion of buildings with excessive dampness. This may lead to increased risk of allergies and asthma of home occupants. Although the evidence is currently limited, climate change through increased temperatures and humidity may also increase the atmospheric concentration of fungal spores, which combined with an earlier seasonal start and increased allergenicity of fungal spores may further increase the risk of allergies and asthma and increase the overall disease burden of allergic diseases and asthma. However, the evidence is currently still limited and the magnitude of these effects is unclear.

## Allergy and asthma in the tropics

For many years, most of the information and research in the field of allergy came from developed countries in both the northern and southern hemispheres. In contrast, knowledge about allergies and asthma in low and middle income countries in the tropical areas is relatively low; nonetheless, more insights on the burden, triggers, and management of these have increased over the last decades.

The Tropics is a very dynamic region surrounding the Equator with a vast biodiversity and climatic variability, ranging from warm to hot and moist year-round. Tropical rainforests have their rainfall equally distributed throughout the year. In addition, climate change could have a big impact on the region, having an influence not only on the climate of the Tropics by itself but also on its biodiversity. The northern limit of the Tropics is the Tropic of Cancer, whereas the limit in the southern hemisphere is the Tropic of Capricorn (23 ° 26’ 16” north and 23 ° 26′ 16″ south, respectively). The Tropics is also called the *tropical zone* or the *Torrid Zone* due to very hot usually dry temperatures. All twelve months have mean temperatures above 18 °C (64 °F). In the Tropics the sun’s rays are hitting the planet exactly perpendicular to its surface. The Tropics has clear distinguishable features from the other climatic and biomatic regions of the Earth, the middle latitudes, and the Polar Regions on either side of the equatorial zone. Therefore, different environmental factors, such as infections (e.g., parasitic infections), type and duration of allergen exposure, and disease expression can be found in patients from the tropical zones as compared to those from the other parts of the globe. In general, allergic diseases and asthma are underdiagnosed and undertreated, and since most of the tropical countries are non-affluent, many patients with allergies and asthma do not have access to appropriate diagnosis, care, and medications, or else they can hardly afford the cost of medications. Whereas the burden of allergic diseases and associated diseases has increased, the number of healthcare professionals trained in the diagnosis and treatment of allergy are very few in proportion. As a result, a large number of patients are undiagnosed or undertreated. While many developing countries have few or no allergy-trained physicians to treat millions of allergy and asthma sufferers, even in developed countries, many highly sophisticated areas have a shortage of allergists [240].

### Africa

The International Study of Asthma and Allergy in Childhood (ISAAC) series provided new information concerning the prevalence of asthma, allergic rhinitis, and eczema in tropical countries. In Africa and as a part of the ISAAC Phase I study, only six African countries were involved (Algeria, Tunisia, Morocco, Kenya, South Africa, and Ethiopia), three of them located in the Tropics (Kenya, Ethiopia, and South Africa). Phase III, conducted 5–6 years later, enrolled 22 centers in 16 countries including the majority of the centers involved in Phase I and new centers in Morocco, Tunisia, Democratic Republic of Congo, Togo, Sudan, Cameroon, Gabon, Reunion Island, and South Africa (6 out of 16 belong to the tropical zone). There were considerable variations between the various centers in Africa on the prevalence of the main symptoms of the three conditions: wheeze (4.0–21.5 %), allergic rhinoconjunctivitis (7.2–27.3 %) and eczema (4.7–23.0 %). There was also a large variation between countries and between centers within the same country. Several centers, including Cape Town (20.3 %), Polokwane (18.0 %), Reunion Island (21.5 %), Brazzaville (19.9 %), Nairobi (18.0 %), Urban Ivory Coast (19.3 %) and Conakry (18.6 %), showed a relatively high asthma symptom prevalence, similar to those in Western Europe. There were also a number of centers showing high symptom prevalence for allergic rhinoconjunctivitis (Cape Town, Reunion Island, Brazzaville, Eldoret [Kenya], Urban Ivory Coast, Conakry, Casablanca, Wilays of Algiers, and Sousse) and eczema (Brazzaville, Eldoret, Addis Ababa, Urban Ivory Coast, Conakry, Marrakech, and Casablanca). In Africa, 13 countries located in the tropical area took part of the ISAAC Phase III Survey [241].

There is evidence that asthma is increasing in prevalence and severity in Africa. The ISAAC study has shown that there is also an increase in the prevalence of allergic rhinitis and eczema. Despite the fact that previous studies have suggested that the prevalence of atopy in West Africa is low, more recently it has been shown that allergic sensitization to house dust mite and other allergens such as cockroach are risk factors associated with asthma in Ghana [242]. As in other parts of the world the upward trend in allergic diseases has been associated with an increase in urbanization; the increased urbanization and change in lifestyles in the Tropics are possible among the factors associated with the increase in the prevalence of allergy and asthma. In the urban population in Ethiopia, *Dermatophagoides pteronyssinus* skin sensitization was a risk factor related to wheeze and was stronger than in the rural areas. *D.pteronyssinus* sensitization was common in rural areas but was not a risk factor for wheezing, possibly due to the high prevalence of parasitic infections in these areas. This study suggests that an increased prevalence of parasite infection may prevent the development of asthma in sensitized atopic individuals [243].

In contrast, children from Kenya living in rural areas had a lower percentage of body fat, smaller and fewer skin test responses to allergens, a higher prevalence of IgE antibodies to *Ascaris*, and 10-fold higher total IgE compared to urban children in Kenya. In the urban area of Kenya, there was a strong correlation between exercise-induced bronchospasm (EIB) and atopy determined both by IgE antibodies and positive skin prick tests. By contrast, in the rural area, none of the 13 children with EIB were skin-test positive (vs 13/109 of children without EIB) [244].

Besides the above information regarding the upward trend in the prevalence of asthma and allergies in Africa as reported in the ISAAC study, another study has confirmed not only the increased prevalence of asthma but also increased allergen sensitization. Two surveys conducted using the same methodology 10 years apart (1993 and 2003) among schoolchildren aged 9–16 years attending urban rich (UR), urban poor (UP), and rural (R) schools were performed in Ghana. The prevalence of both exercise-induced asthma (EIA) and sensitization approximately doubled over the ten-year period among Ghanaian children and adolescents irrespective of location, with both asthma and atopy being more common among the UR than the UP and R children [245]. Moreover, students from affluent schools had higher levels of IgE to mites compared to those non-affluent and suburban/rural students from Ghana [246].

In a survey conducted in Kigali (the capital of Rwanda) and in Huye District (a rural area located in southern Rwanda), airflow obstruction was found in 256 participants (14 %). Of that number, 163 (8.9 %) subjects were asthmatics and 82 (4.5 %) had chronic obstructive pulmonary disease (COPD). 584 subjects (26.5 %) had positive skin-prick tests, and house dust mite and grass pollen mix were the main allergen sources found. The major risk factors for asthma were allergy, female gender, and living in Kigali [247].

**Fig. 4:**
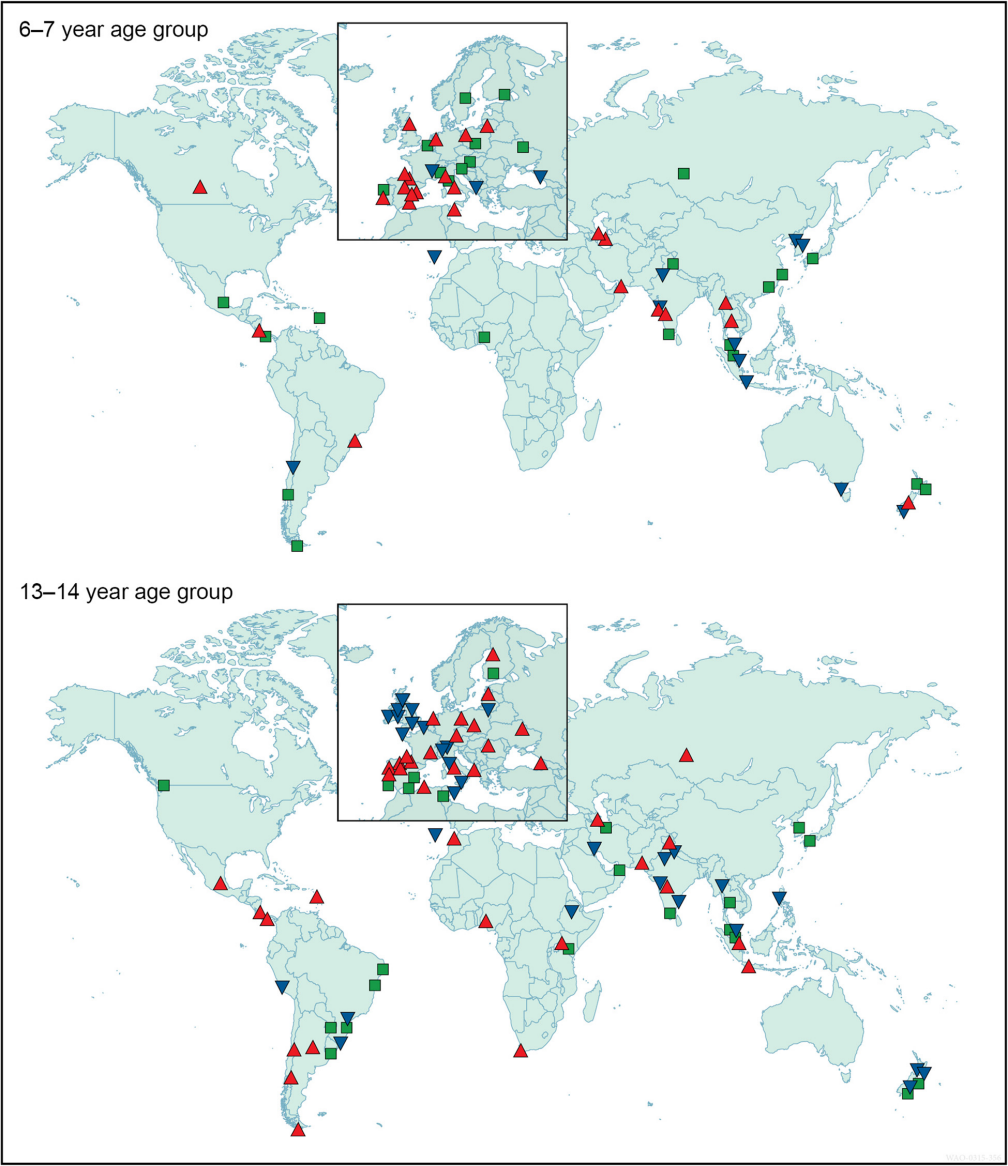
Blue triangles identify locations where prevalence was reduced by at least 1 standard error (SE) per year. Green squares identify locations where there was little change in prevalence (i.e., change of less than 1 SE per year). Red triangles identify locations where prevalence increased by at least 1 SE per year. Reused with permission from Elsevier [141]

All of this information confirms that asthma and allergic sensitization are becoming more prevalent in many African countries and that this upward trend is mainly related to sensitization to house dust mite, tobacco smoking, and living in urban affluent westernized locations. The high prevalence of severe asthma found in Africa in the ISAAC study (Fig. 4) suggests that limitations in getting access to care, as well as the availability and affordability of medications, can contribute to severe forms associated with non-treated patients, as defined by the World Health Organization (WHO). A better socio-economic situation could help to treat those non-treated patients and decrease the severity of asthma and allergy [248, 249].

The tropical environmental factors which may cause allergy include food items, animals, birds as well as the house dust mites and stinging insects (bees and wasps) that are present, to varying degrees in all continents. Plant derived allergen sources span trees, grasses, and weeds which can be unique to local environments. There is also sensitization to airborne mold spores. Tropical dietary habits are influenced by the availability of edible grains, plants, fruits and even insects. These are potential sources of allergen sensitization and due to their habitat specificity are likely to display regional peculiarities. This discussion of allergen sensitization in tropical Africa is limited to the observations that have been made in Zimbabwe, a Subtropical African country. Examples from some neighboring countries are incorporated [141].

**Fig. 5:**
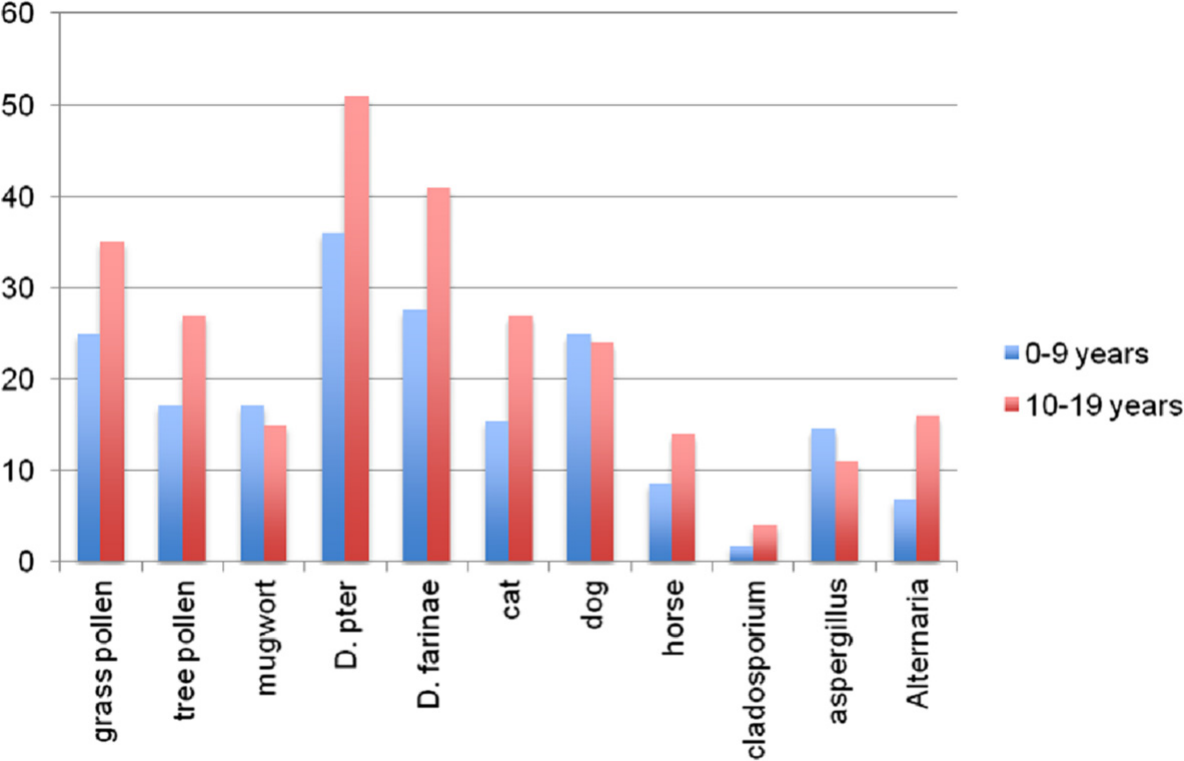
Inhalant allergen sensitization pattern in children and adolescents in Zimbabwe. The vertical scale represents a percentage of some 987 sensitized patients. Source: Elopy N Sibanda

The ISAAC was limited to a handful of centers in tropical Africa [141, 243]. In the limited number of centers, the study reported prevalence rates of wheezing, allergic rhino-conjunctivitis or eczema to range between 4 % and 27 % amongst 12–14 year olds in 22 centers selected from 16 African countries. These findings debunked the commonly held perception that allergies are infrequent in Africa. Whereas the presence of wheezing, rhinitis, conjunctivitis and dermatitis points towards allergen sources as potential triggers, there remains a dearth of studies that identify triggering specific allergen sources. There have been limited attempts to correlate allergen sources with clinical disease manifestations. In Zimbabwe the predominant allergen sources are house dust mites (*D. pteronyssinus* and *D. farinae*), grass pollen, animal hair and molds [250–252] (Fig. 5).

#### House dust mites

House dust mite sensitization is ubiquitous, occurring worldwide in both temperate and tropical environments. House dust mites are the predominant sources of inhaled allergens in Zimbabwe. Dust mite sensitivity is associated with asthma and rhinitis. Approximately half (52 %) of all the patients with asthma or allergic rhinitis and conjunctivitis are sensitized to house dust mites. While house dust mite sensitization is ubiquitous, there are significant differences between specific house dust mite component molecules that elicit allergic diseases in this tropical country compared to temperate climate, with Austria used as an example. In Zimbabwe, the major allergenic molecules are the Der p 1 (80 %), Der p2 (45 %), Der p 7 (35 %), Der p10 (55 %). This allergenic molecule pattern differs from the observations in Austria, where there was no (0 %) sensitization to Der p 7. Similarly Group 10 tropomyosins are comparatively high (55 %) in Zimbabwe compared to the Austrian population (10 %). Higher prevalence of Group 10 tropomyosin sensitivity has also been reported from tropical Japan (80 %). Tropomyosins that are structurally similar to Der p10 are found in crustaceans (crab, lobster, and shrimp) and insects (cockroaches). The presence of crustaceans and insects is influenced by climate and geography. Significant differences in the IgE-binding frequency of Der p 11 (the mite paromyosin) have also been observed between temperate (Austria 12 %, France 5 %, Italy 7 % and Sweden 11 %) and tropical (Zimbabwe) countries. In Zimbabwe, the IgE binding frequency of Der p 11 was much higher (36 %) and was associated with atopic dermatitis [253].

A careful analysis of specific allergenic molecules reveals geographical variations in the prevalence of sensitization and allergic reactivity to specific molecules (Table 5). Different house dust mite molecules can cause similar respiratory symptoms in different climatic environments. There are implications on the diagnosis and management of allergic conditions. The use of house dust mite extracts in skin prick tests and/or in serological diagnostic techniques only provides a general guide to the sensitization patterns. Component resolved molecular detection techniques are required to identify specific sensitivity patterns and regional specificities and ultimately to tailor specific immunotherapy interventions.

**Table 5:** Comparison of sensitization to house dust mite molecules between a tropical and temperate country. Source: Elopy N Sibanda

	MW	Austria	Zimbabwe
rDer p1	26	96 %	80 %
rDer p2	10.7	15 %	45 %
rDer p7	23.7	0 %	35 %
rDer p10	36	10 %	55 %

#### Cockroaches

Sensitization to cockroach allergens is common. Cockroach allergy is a recognized cofactor in both asthma and rhinitis and increases the severity of both. Asthma severity is greater in cockroach sensitive subjects and is associated with other atopic conditions such as dermatitis. Patterns of sensitization to cockroach allergens were noted to be variable in two neighboring tropical countries (South Africa and Zimbabwe). Patients who live in cities that are located at higher altitudes (Harare, Zimbabwe and Pretoria, South Africa) have predominant *Blatella germanica* sensitization, whereas patients from coastal Cape Town and Durban (both in South Africa) have a mix of *B. germanica, Periplaneta americana* and *B. orientalis* [254]. The results show that sensitization patterns can vary within the same climatic zones and are influenced by factors such as proximity to the sea or altitude. However the relative effect of sensitization to one or the other types of cockroaches in this region has not been investigated.

#### Pollen

The predominant grass pollen source is Bermuda grass *(Cynodon dactylon)* which belongs to the *Poaceae* family of grasses in Bermuda and Southern Africa. This is a typically tropical grass whose optimum growth occurs at temperatures between 24 and 37 °C and is most abundant at latitudes between 30 °N and 31.4 ° + 7.5 °S. In view of its value in soil erosion control, this grass variety was widely planted alongside roads and school fields across Zimbabwe and has become widely abundant. Bermuda grass is implicated in seasonal exacerbations of allergic rhinitis, asthma and allergic conjunctivitis.

Maize pollen *(Zea mais)* another Type 1 grass is a common source of respiratory allergens in Zimbabwe. Like Bermuda grass, maize belongs to the Poaceae family of grasses and is an important cereal crops. It is widely cultivated in tropical climates and is responsible for respiratory and to a lesser extent food allergy.

#### Food allergy

**Fig. 6:**
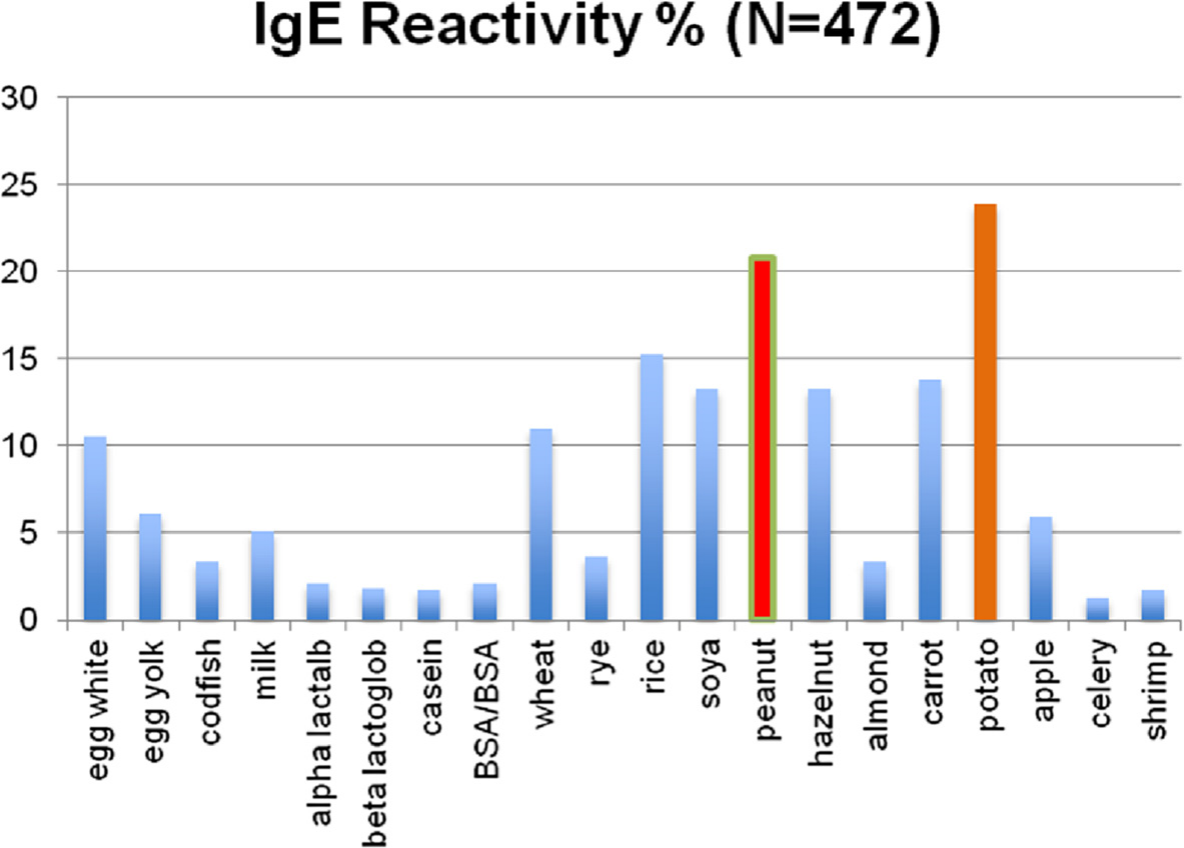
Allergen-specific IgE reactivity in patients presenting to the Asthma Allergy and Immune Dysfunction Clinic in Harare. Source: Elopy N Sibanda

Food allergen sources that are prevalent in Zimbabwe reflect the dietary habits of the people. The full repertoire of these is limited by the availability of both skin prick testing extracts and locally relevant test panels. The reported results reflect a concordance of both skin prick testing and serological assays of IgE mediated reactivity. The predominant food allergen sources are potato (24 %), peanut (21 %), rice (15 %), carrot (14 %), soya (13 %), hazelnut (13 %) and wheat (11 %) (Fig. 6). These allergen sources have been relatively recently introduced and became popular components of the diet of the indigenous Zimbabwean population in the last 50 years. A closer evaluation of peanut sensitization was conducted in view of world reports of their association with anaphylaxis and mortality.

A unique observation is that despite high levels of sensitization to both potatoes and peanuts there are no reported cases of severe peanut induced reactions or anaphylaxis. Peanut sensitization was demonstrated using skin prick extracts and confirmed by allergen specific IgE detected using the ImmunoCAP technique. Allergen specific IgE antibodies were also confirmed using a Western blotting technique. All the extracts and serological reagents used were from commercial sources and were the same that identified sensitization in patients with severe reactions and anaphylaxis in temperate countries. Further testing using resolved allergen molecular components confirmed sensitization to rAra h 2, rAra h 1, rAra h 3, nAra h 6 and rAra h 9. The levels of IgE antibodies to Ara h 1 Ara h 2 were sometimes greater than 15 kU_A_/L. These allergens and this level of sensitization is often associated with severe reactions and anaphylaxis in temperate countries, but not in Zimbabwe, suggesting unexplained peanut tolerance. Similar tolerance has been reported from Nigeria [255]. The authors speculate that it is the method of peanut preparation and the timing of its introduction to the diet that produces tolerance as opposed to allergy. There may well be other explanations.

### The Americas

The tropical area in the Americas extends from Mexico in the Northern hemisphere to a line that crosses Paraguay and the South of Brazil. Most of the Latin American countries are part of the Tropics except Argentina, Chile, and Uruguay. The Tropics also includes the southern half of Mexico, Central America, and the Caribbean. The tropical South America sub-region comprising 10 countries (Colombia, French Guiana, Suriname, Guyana, Venezuela, Ecuador, Peru, Bolivia, Paraguay, and Brazil) represents the greatest concentration of tropical rainforest in the world. The Amazonian tropical rainforest is considered to be the world’s richest ecosystem in terms of biodiversity. The rainforest in South America is approximately 885 million hectares in the Amazon Basin and another 85 million hectares in the Orinoco and Paraná watershed complex. The total land area of tropical South America is 1,387 million hectares. The temperature in a rainforest rarely is higher than 93 °F (34 °C) or drops below 68 °F (20 °C), and the average humidity ranges between 77–88 %. The rainfall is often more than 100 in. a year. Terrestrial biodiversity (degree of variation of life) tends to be the highest at low latitudes near the Equator. The tropics in Mexico, Central and South America are extremely rich in terms of genetic variation, species variation, or ecosystem variation. For instance, Mexico is home to 10–12 % of the world's biodiversity and is one of the 17 M diverse countries as well as other 7 Latin American countries (Brazil, Bolivia, Colombia, Costa Rica, Ecuador, Perú, and Venezuela).

The ISAAC Study in Latin America has shown that the prevalence of lifetime asthma ranged from 1.2–33.1 %, whereas current wheezing was from 3.9 to 30.8 %. An important proportion of centers (55 %) reported a prevalence of asthma symptoms over 15 %. There was no significant correlation between prevalence of asthma symptoms and the latitude, altitude, or tropical setting. It is suggested that ecological interactions, probably typical for each locality, may be the main determinants for the large variability of asthma prevalence in Latin America [256].

Regarding allergic rhinitis, 93,851 children (6–7 years old) from 35 centers in 14 Latin American countries, and 165,917 adolescents (13–14 years old) from 56 centers in 17 Latin American countries, were enrolled. The mean prevalence rate of current rhinoconjunctivitis was 12.7 % in the region while the highest prevalence was found in Caracas (Venezuela) – 21.2 % of the schoolchildren. The mean prevalence rate of current rhinoconjunctivitis was 18.5 % and ranged from 7.1 % in Cuernavaca (México) to 45.1 % in Asunción (Paraguay) for the adolescents. In general the prevalence of rhinitis-related symptoms was higher among the Spanish speaking centers. As it happens for asthma, a great variability in the prevalence was also found for allergic rhinitis including differences observed even in centers from the same country [257].

The prevalence of eczema in tropical Latin America is also very high compared to other regions and countries that participated in the global survey. The mean prevalence of current flexural eczema in schoolchildren was 11.3 %, ranging from 3.2 % in Ciudad Victoria (México) to 25.0 % in Barranquilla (Colombia). For adolescents, the prevalence varied from 3.4 % in Santo André (Brazil) to 30.2 % in Barranquilla (mean prevalence, 10.6 %) [258].

Atopy is very common among children and adults with asthma and/or allergic rhinitis. A study looking at the prevalence of sensitization to 6 different mites species (*Dermatophagoides pteronyssinus*, *D. farinae*, *Blomia tropicalis*, *Chortoglyphus arcuatus*, *Lepidoglyphus destructor,* and *Aleuroglyphus ovatus*) in 297 children and adults with asthma from 7 cities in 5 Latin American countries (most of them tropical cities – Bogotá, Caracas, Cartagena, São Paulo, and Mexico City) was performed. Sensitization to *D. pteronyssinus* varied from 60.7 % in Cartagena to 91.2 % in São Paulo; to *D. farinae* from 53.3 % in Córdoba to 97.2 % in Caracas; to *A. ovatus* from 26.6 % in Bogotá to 71.2 % in São Paulo; to *B. tropicalis* from 46.5 % in Mexico City to 93.7 % in São Paulo; to *C. arcuatus* from 33.3 % in Mexico City to 75 % in São Paulo; and to *L. destructor* from 30 % in Mexico City to 76.2 % in São Paulo. The studies carried out in São Paulo and Córdoba were confined to children and thus could be compared; there was a significantly higher prevalence of cutaneous sensitivity to mite allergens in the children of São Paulo (in the Tropics) than in those of Córdoba (*p* < 0.001 for all mite species). Cutaneous sensitivity to mite allergens is very common in young and adult asthmatics in Latin America, in areas both at sea level and at high altitudes [259].

More recently, it has been shown that 76 % of patients with respiratory allergy are sensitized to aeroallergens, especially mites in Caracas, a tropical city [260]. Moreover, anaphylactic reactions after the ingestion of mite-contaminated pankakes has been also described in Venezuela indicating that not only the inhalation of mite particles but also ingestion can elicit oral mite allergic reactions [261]. An online registry of anaphylaxis has been developed and 191 patients registered from Latin America and Portugal. Anaphylactic reaction to foods (36.1 %), drugs (27.7 %), and insect stings (26.2 %) were analyzed. The most common symptoms during an acute episode were cutaneous (94.2 %) as well as respiratory (78.5 %). Most patients were treated in emergency setting, yet only 34.6 % received injective epinephrine and 14.3 % had to be hospitalized. The management of anaphylaxis needs to be improved in the region [262].

Environmental features in the tropics in Latin America have to be taken in account regarding risk factors and potential pathogenic agents. *Ascariasis* infections are important health problems in low-income settings such as those located in the tropics where environmental conditions also promote the perennial co-exposure to high concentrations of domestic mite allergens. Experimental evidence of a high cross-reactivity between the allergenic extracts of these invertebrates, involving well-known allergens such as tropomyosin and glutathione transferasas has been described [263]. *Ascaris lumbricoides* induces a Th2 response and specific IgE synthesis in humans; in addition, *Ascaris* tropomyosin is cross-reactive to mite tropomyosin and can induce wheal and flare reaction in skin prick tests and histamine release from basophils. There is indirect evidence suggesting that the clinical impact of these findings may be important [263].

In a recent study performed in the north of Brazil, it was found that children of more educated mothers, living in improved environmental conditions, and with a low burden of infection were significantly more likely to have the responsive phenotype. The so-called responsive phenotype was significantly associated with an increased prevalence of atopy but not asthma. A better understanding of the underlying immune mechanisms of the hygiene hypothesis in urban Latin America is needed [264].

Early life sensitization to aeroallergens, presence of atopic dermatitis or allergic rhinitis, maternal smoking during pregnancy and children's environmental exposure to tobacco smoke, lower respiratory tract infections with respiratory syncytial virus and potentially with other viruses including rhinovirus and metapneumovirus, exposure to air pollutants, several perinatal factors other than maternal smoking, are among the factors associated with an increased risk for the development of chronic diseases like asthma and atopy [265].

### Conclusions

Allergen sources predispose to allergic diseases in genetically predisposed individuals worldwide. This is true and expected for allergen sources that are geographically demarcated such as food items, tree and grass pollen. There are also significant differences with ubiquitous allergen sources such as house dust mites and cockroaches. The molecules responsible for allergy vary geographically. Tropomyosins contribute more to allergy in the tropics (Japan, Zimbabwe) than they do in the temperate regions. While the allergen sources may be the same, the allergen molecules differ, necessitating component resolved diagnosis and targeted treatment for house dust mite allergy. Cockroach allergy varies with altitude and proximity to the sea. The *germanica* species thrive in high altitude whereas the *P. americana* are in low altitude. The lesson to be learned is that while major allergen sources may be ubiquitous, local variations impact on accurate diagnosis, prevention and treatment of resultant allergic diseases.

## New emerging factors with migration

The prevalence and impact of allergy have been associated with environmental and lifestyle changes accompanying the continuous process of urbanization and globalization. Changing environmental factors are most likely important in explaining the trend of asthma prevalence worldwide. Asthma is the result of environmental factors on a genetically susceptible subject. Socio-economic background, family size, urban dwelling, farm exposure, infections, diet, obesity, drugs, tobacco smoke exposure and indoor and outdoor air pollution are among the factors associated with atopic disease [266].

Comparative studies have demonstrated that asthma and related allergic disorders are more common in westernized or urbanized societies than in rural or developing countries [267]. Asthma prevalence appears to have peaked or reached a plateau in countries with a high prevalence but is still rising in some. Urbanization and economic development cannot explain the reason for a lower prevalence of asthma in cities such as Singapore, Seoul, and Hong Kong as compared to the United Kingdom and Australia [268].

Rural environment, farming exposure to a microbial diversity and animals, and consumption of unpasteurized farm milk have protective roles for asthma and allergic disorders [269–271]. Exposure to poultry and livestock in a farm environment could reduce the prevalence of hayfever and atopic sensitization in children born and raised in farms [272, 273]. A study of the differential effects of farm-associated exposures on specific asthma-related health outcomes demonstrated that the asthma-protective effect of being raised on a farm could be attributed to pig farming, feeding silage, child’s involvement in haying, farm milk consumption, and regular stay in animal sheds and barns. The microbial compounds (extracellular polysaccharide, endotoxin and glucans) measured in mattress dust did not explain the inverse relation of asthma prevalence [274].

The disparities in asthma prevalence and morbidity among the world's geographic locations are more likely to be associated with environmental exposures than genetic differences. In Malaysia asthma and asthma-related symptoms occurred more frequently in urban than in rural areas, and that difference correlated with environmental risk factors such exposure to dust mites, high levels of vehicle emissions, and a westernized lifestyle. In urban areas, biomass fuels have been widely replaced by cleaner energy sources at home, but in developing countries, coal is still a main fuel for heating in winter and cooking. Among the poor, cooking devices that are placed in inadequately ventilated spaces have direct health impact [275, 276]. A study investigating the annual respiratory symptoms of 3,049 Japanese students from 8 urban and rural areas showed a positive association between regional NO2 levels and asthma prevalence [277]. Moving from traditional/rural to modern/urban societies could explain the increase in asthma prevalence in developing countries. Rural areas tend to have a low asthma prevalence protected by factors associated with a traditional rural lifestyle [278–280].

Potential risk factors for asthma related to urbanization have been identified: reduction in the frequency of infections; reduction in family size; increasing vaccine coverage; use of antibiotics; increases in environmental pollution and household exposure to allergens; changes in diet, lifestyle and socioeconomic aspects [281]. Acquiring allergy is influenced by the age at the time of immigration. Migrants, in general, are more prone to the development of allergies than the local population [282].

The prevalence of both EIB has approximately doubled over the 10-year period amongst 9- to 16-year-old Ghanaian children irrespective of location, with EIB being more common among the urban than rural children [245] (Table 6). The overall reported prevalence of the CNCDs (diabetes mellitus, hypertension, cardiovascular disease, asthma, epilepsy, depression and mental illness) was found to be 8.9 % in Ethiopia. The specific observed prevalence of asthma was 1.5 % similarly to the rate found 15 years ago in rural Ethiopia [292].

**Table 6:** Prevalence of asthma in urban and rural areas of developing countries [245, 283–291, 295]

Country	Author, year	Urban	Rural
South Africa	Steinman 2003	33	17
Zimbabwe	Keeley 1991	5.8	0.1
Ethiopia	Yemaneberhan 1997	3.6	1.6
Ghana	Addo Yobo 1997	4.7	2.2
Ghana^a^	Addo-Yobo 2007	8.3	3.9
Kenya^a^	Ng’ang’a 1998	22.9	13.2
Keny^a^	Odhiambo 1998	9.5	3.0
Saudi Arabia	Hijazi 1998	14.9	5.4
South Africa^a^	VanNiekerk 1979	3.17	0.14
Peru	Robinson 2011	12	3
Brazil	Solé 2007	16.7	15.3

^a^Exercise-induced bronchospasm

Atopic sensitization to indoor allergens such as house dust-mite and cockroach is strongly associated with asthma symptoms among urban compared to rural Ghanaian children [293]. A diversity of factors may influence the lower prevalence in rural areas [293]. Although the exact protective environmental factors in the rural region remain to be defined, there have been many studies suggesting that early exposure to microbes or microbial products may play a role in modulating the immune system so as to reduce the future risk of asthma and allergies [294]. Asia-Pacific is one of the most densely populated regions of the world and is experiencing rapid economic changes and urbanization. Although the prevalence of asthma and allergies remains relatively low in Asia compared with other regions, the rapid modernization of many cities in South Asia has an increased the prevalence and severity of asthma [268].

Urbanization is a complex array of changes that modify the living environment and human behaviors, which can be significantly associated with current asthma symptoms. Higher asthma prevalence has been found in Ecuadorian communities with a higher socioeconomic level and a more urbanized lifestyle [281]. Robinson et al.*,* studied 13–15-year-old adolescents living in two coastal regions of Peru: Pampas de San Juan de Miraflores, a *peri-urban* shanty town in Lima with high population density centered on a heavy traffic route, and 23 rural villages with little traffic outside Tumbes city in northern Peru. Significantly higher current asthma symptoms (12 % vs 3 %), current rhinitis (23 % vs 12 %), eczema (12 % vs 0.4 %), atopy (56 % vs 38 %) and higher exhaled nitric oxide (eNO) values were observed in urban adolescents [290].

The prevalence rates for asthma in Kenya, defined as “attacks of shortness of breath with wheeze”, were 9.5 % for urban and 3.0 % for rural children (odds ratio (OR) urban versus rural: 3.42; 95 % confidence interval (CI): −1.96–5.91) [290]. The prevalence rates of exercise-induced bronchospasm (EIB) were considerably higher in urban than in rural African children [286]. Methodological differences (age groups, definition of current asthma, questionnaires) likely explain the differences observed in epidemiological studies. Urban–rural differences in diet, the frequency and nature of childhood infections, exposure to industrial and motor vehicle emissions have been implicated. In addition aeroallergens distribution may vary accordingly with the environment [286–289]. Higher rates of current symptoms of asthma and rhinitis but not eczema had been reported for adolescents living in two urban centers of ISAAC study in Brazil as compared to those living in rural areas [295].

### Conclusions

Migration involves exposure to a new set of pollutants and allergens as well as changes in lifestyle conditions, all of which are likely to affect migrants' health [159]. Undoubtedly, migration from rural regions to urbanized centers is accompanied by an increase in prevalence rates of asthma in different regions of the world. The reasons of this trend are diverse and there is interaction between environment risk factors and a genetically susceptible host. Research efforts must achieve sustainable results on prevention, diagnosis and treatment of this most prevalent chronic disease both for populations in developing countries and for immigrants from such countries to atopy prevalent ones [159, 266].

## The role of air filtration as an environmental control measure for allergic disease

In the treatment of asthma and allergies, the most effective environmental remediation strategies are multifaceted interventions [296–299]. Control of the environment requires attention to exposures that originate from both the outdoor and indoor environments. Avoidance of environmental factors that provoke asthma, where feasible, is a logical way to improve asthma-related health and to minimize the need for long-term use of asthma medications. Air filtration is frequently recommended as a component of environmental control measures for patients with allergic respiratory disease [297–300].

The association between preventive asthma care and comprehensive environmental control practices was examined in a review of 3,727 adults with asthma by Roy and Wisnivesky [301], using data from the Four-State National Asthma Survey. Comprehensive management was defined as the implementation of combinations of at least five of eight measures. Air filtration was found to be the fourth (27.4 %) most commonly implemented strategy, preceded only by no smoking (80 %), no pets (53.9 %), and washing sheets in hot water (43.2 %), and followed by pillow covers (23.7 %), mattress covers (23.4 %), no carpets (14.5 %), and use of a dehumidifier (13.8 %) [301]. A similar study in the pediatric population found the same rate of air filtration (27.4 %) use in the households of asthmatic children [302].

Residential air filtration can be provided by whole house filtration (WHF) via the home’s heating, ventilation, or air conditioning system (HVAC) by portable room air cleaners (PRACs), or a combination of the two [302]. Use of high-efficiency particulate air (HEPA) filters reduce airborne allergens in the indoor environment and may provide clinical benefits for patients with respiratory allergies [130].

The role of air filtration in disease prevention has been studied for long time but it continues to be debated. Several investigations have demonstrated that indoor air cleaning devices can reduce concentrations of asthma triggers in indoor air [297, 303–317]. Recently, Brown et al. [297] evaluated the performance of different grades of filters in a modeling, identifying filters to be effective at reducing airborne asthma triggers by at least 50 %.

In a study conducted by Du et al. [303], using HEPA filters reduced particulate matter by 69–80 % in the homes of children with asthma. Previously it was demonstrated that substantive reductions in fine particulates were attained in ambient space with the use of enhanced efficiency WHF [304].

Macintosh et al., [305] also found WHF to be effective in removing fine particles from indoor air. Lanphear et al.*,* [306] conducted a double-blind randomized control trial of 215 asthmatic children with known second-hand tobacco smoke exposure. The intervention group received 2 HEPA filters and the control group received 2 sham air filters. It was demonstrated a reduction of airborne nicotine particles and in the number of unscheduled asthma visits in a 12-month follow up period in the intervention group. There was not a difference in serum or hair cotinine between the two groups [306].

Recent investigations suggest that HEPA air filtration reduces mold levels in hospital clinical units [307] and reduces fine PM (diameter, <2.5 μm) exposure during forest fires and residential wood burning [308]. Lingell et al. [309] conducted an environmental remediation in a school in Finland with poor air quality and high levels of mold. After installing a ventilation and exhaust system, there were lower levels of mold but this was not associated with fewer respiratory complaints.

In a study by Wood et al., excluding cats from the bedroom and using HEPA filters were found to be effective in reducing cat allergen levels; however, there was no reported improvement in asthma or allergic rhinitis symptoms [310]. Van der Heide et al., [311] conducted a 3-month HEPA filter intervention study in 20 pet-sensitized children with asthma and demonstrated significant reductions in bronchial hyperresponsiveness but no differences in symptom scores or medication use in pet-sensitive asthmatic children. These results were not replicated in a similar study [312]. Reisman found reductions in asthma/rhinitis symptoms during active HEPA filter use [313].

Morgan et al., [314] as part of the Inner-City Asthma Study Group, conducted a randomized controlled trial of comprehensive ECPs in 937 children with atopic asthma. Interventions included the use of a HEPA room air cleaner in the child’s bedroom if the child was exposed to ETS, sensitized and exposed to cat or dog allergens, or sensitized to mold. The intervention group reported significantly fewer symptoms of asthma during the intervention year and the follow up year [314].

A review of 10 randomized controlled trials by McDonald et al., [315] reported that HEPA air filtration was associated with symptom reduction. However, caution was suggested by methodological flaws in most of the studies. Morris et al., [316] found significant reductions in allergic rhinitis symptoms in ragweed-sensitive children and adults. Brehler et al., [317] reported improvement in seasonal allergic rhinitis symptoms with air filtration through a wall-mounted filter. Sublett et al., [130], in his air filtration review, concluded that there is sufficient evidence to suggest that air filtration decreases allergic respiratory disease progression. Studies support multiple interventions, including air filtration, as methods to improve outcomes in the treatment of allergic respiratory diseases. In addition, considering the enormous burden of asthma in terms of costs and adverse health effects, air filtration is a relatively low-cost component of efforts to reduce allergens in homes.

## Cold weather

### Positioning of “cold” within the context of global warming

Cold air has well defined effects on the lungs both in terms of the individual asthmatic patients and of the respiratory health of susceptible populations. However, when discussing climate changes allegedly due to anthropogenic activities, the emphasis is placed on global warming usually in conjunction with air pollution. The general perception is that the overall rise in mean temperature of 2 °C above preindustrial levels associated with unprecedented high CO2 of 396 · 8 parts per million in early 2013 is creating periods of draught, wildfires and enhanced harmful effects of air pollution affecting more severely the poor and underprivileged layers of human society [318]. Not surprisingly effects of cold climate remain out of the focus of the media unless spells of unusually cold weather sweep through a local area. However, noteworthy features of climatic change are unstable weather conditions associated among other things with extremely cold periods of increasing frequency and duration. An ongoing project in the European Union with the acronym PHASE (Public Health Adaptation Strategies to Extreme weather events) is specifically meant to look at the interaction between air pollution and extreme weather events (e.g. heat waves and cold spells) [319]. Cold weather episodes in turn introduce an element of uncertainty in society as to the reality of climatic warming as a global phenomenon. The skepticism that global warming is real increases during cold weather spells as evidenced by analysis of US media coverage and opinion polls since 1990; conversely, concerns that global warming is imminent and menacing surge when average temperatures are soaring [320].

### Cold air alone or in combination with other factors

Temperatures fluctuate around the year depending on the latitude of a given region, its altitude, and the proximity to large water reservoirs. It is common knowledge that the winter season in the higher latitudes is the worst part of the year for many patients with chronic respiratory diseases. To obtain information on the extent and severity of asthmatic symptoms during daily life in winter, a simple questionnaire was sent to 57 asthmatic patients and a control group of 180 age-matched men and women in Göteborg (Sweden). The average winter temperature there is at about the freezing point. About two-thirds of the asthmatic patients reported cold to be a factor causing breathing difficulties. In 37 %, these symptoms made the patients avoid going out during the winter [321].

However, there is no “universal” numerical value of air temperature that can be accepted as cut-off point for “cold”. It is rather the magnitude of downward temperature change below the mean seasonal range for a given area that challenges the adaptive ability of people. Mortality increased to a greater extent with given fall of temperature in regions with warm winters, in populations with cooler homes, and among people who wore fewer clothes and were less active outdoors [322]. As adaptive capacity shrinks with age, it is the elderly who are mostly affected. Thus, it has been documented that cold temperatures are associated with a 3–4 % increase in daily mortality and hospitalization for respiratory causes in the population over 75 years old for each degree Celsius decrease in minimum temperature or minimum apparent temperature (defined as a combined indicator of temperature and humidity above a city specific threshold level ranging from 23-29 degrees Celsius) [323, 324].

The effect of cold temperature is modulated by other ambient conditions too. As an example, cold damp air was reported by asthmatic patients to cause more symptoms than cold dry air, while the control group reported very few respiratory symptoms [325]. Modeling of the effect of cold air is rendered much more complex when combinations of several meteorological variables are considered. Such combinations are referred to as “synoptic air masses”, where humidity, visibility, cloud cover, air pressure, wind speed and others are added into the equation and are known to influence mortality and morbidity [187]. It is conceivable, though, that in such sophisticated models specific evidence for respiratory causality is difficult to obtain.

### “Cold” and air pollution

Climate change and air pollution due to anthropogenic activities are intrinsically connected since greenhouse gases and air pollutants originate from the same source - fossil fuel combustion. The bulk of air pollutants are associated with increase of temperature, discussed elsewhere in this monograph. Alternatively, nitrate particles, as well as organic carbon aerosols have a cooling effect on the climate. Sulfur dioxide partly converts to sulfate particles, which also have cooling potential, so they partly react with black carbon, neutralizing its strong warming effect [324]. Also in some cases as in the study of Carder et al., cold temperatures in conjunction with black smoke concentrations increases respiratory mortality [326]. Since extremes of cold and particulate pollution may coexist, for example during temperature inversion, these results may have important public health implications.

### Mechanisms of cold air effects

The respiratory system may be particularly affected by cold air exposure as inspired air has to be conditioned before participating in peripheral lung gas exchange, with an associated loss of heat and water. Furthermore, when the individual exercises, nasal breathing quickly switched to mouth breathing, particularly at minute ventilations above 40 l per minute, with the involvement of intrathoracic airways in this conditioning process [327].

Although exercising in cold air has minimal influence on airways of normal individuals, it can induce a bronchoconstriction in asthmatic subjects and worsen airway obstruction in those with obstructive pulmonary diseases [328]. Winter athletes can be particularly affected by these environmental conditions, and an increased prevalence of airway hyperresponsiveness, asthma and chronic cough has been described in this population [329–331]. Bronchial biopsies of winter athletes have shown evidences of airway remodelling, possibly due to the repeated cold-air and hyperventilation damage to the airways, although more research is needed on this influence on airway function [332]. The mechanism of bronchoconstriction as a response to exercise-induced hyperpnoea, particularly in cold air, has been studied and seems mostly related to an increase in airway fluid osmolarity following hyperpnoea, although heat loss may be a modulator of this response, as well as a possible post-exercise “rewarming” of the airways [333, 334].

Even in normal subjects, cold air can induce changes in the airways. Exposure to cold air can increase the number of granulocytes and macrophages in the lower airways [335]. Furthermore, cold-related impairment of respiratory mucociliary function can inhibit the clearance of pollutants [336]. Finally, in extreme cold temperatures, people tend to gather indoors and crowding can promote the transmission of infectious agents with ensuing airway inflammatory events.

### Role of the upper airways in health and asthma

Breathing cold air has been long recognized to trigger bronchoconstriction in asthmatics. In a classical experiment Shturman-Ellstein et al., demonstrated that if subjects with asthma breathed only through the nose during the exercise challenge, an almost complete inhibition of the post exercise bronchoconstrictive airway response was observed [337]. However, as the nose is serving as outermost filter for the inspired air, it is exposed to environmental hazards with consequent high incidence of morbidity. Adding to this genetic atopic predisposition, it is likely that asthmatic subjects have concomitant rhinitis, which does not allow proper conditioning of the inspired air with negative impact on the asthmatic condition. The cross-talk and interplay between upper and lower airways has been a center point in the philosophy of the Allergic Rhinitis and its Impact on Asthma (ARIA) initiative and has been reconfirmed over the years [338].

The upper airways mucosal structures are particularly sensitive to cold air influences. Challenges with cold dry air have been proposed to assess the state of nasal responsiveness in both allergic and non-allergic rhinitis [161, 339, 340]. This line of research is substantiating the importance of cold weather as trigger in the pathogenesis of rhinitis, which in turn is a recognized risk factor for the development of asthma [341]. Cold weather spells as a characteristic feature of changing climate will need to be considered in assessing the risk for asthma, especially since heterogeneous human populations may adapt differently to them [342].

### Conclusions

There is a need to better define the consequences of repeated exposure to cold air and the mechanisms by which such exposure could modify airway function and affect the outcomes of patients with pre-existing airway disease. This could help promote adequate policy and public health actions to face the incoming challenges. By all means distinction should be drawn between effects to individuals and effects to populations, as populations are heterogeneous in their susceptibility, reversible and irreversible effects should be identified [343].

## Wildfires and asthma

Wildfires include bush fires, vegetation fires, forest fires, and heath and grass fires. These events are widely prevalent around the world and there is evidence that their incidence is increasing. Their cause may be accidental or deliberate, to create deforestation. Increasingly reports consider that the occurrence of wildfires are strongly determined by the incidence of drought and heat waves with climatologists believing that climate change will increase their incidence as the associated droughts and heat waves are expected to increase in frequency and intensity [344].

Over the past 5 years, serious wildfires in Chile [345], Australia [346] and California [347] have reminded the global community of the devastating effects uncontrolled fire may cause. In Europe, there are on average 70,000 forest fires annually. These are an almost annual feature in countries with warmer climates such as Portugal, Greece and France, particularly in the island of Corsica. Apart from obvious burn and other physical trauma caused by wildfires there are also other, less obvious health effects. These have been far less documented than the health effects of heat waves and heat exposure.

Recently, a number of published studies have examined the health effects of wildfires. A study in the United Kingdom (Finlay et al., 2012) [348] set out to collate and review the evidence regarding human health impacts from global wildfire experience. This study identified a wide range of health effects but the respiratory effects were shown to be the most important, both in the short and long term. A further study examining the estimated global mortality attributable to smoke inhalation from wildfires was published by Johnson et al., in 2012 [349]. These and other studies have emphasized the importance of respiratory health problems associated with wildfires.

### Aspects of the epidemiology and pathophysiology of the respiratory effects of wildfire smoke

The particulate matter (PM) in smoke is defined in terms of particle size: 10 μm particles are distributed in the small bronchi while 2.5 μm particles can penetrate the terminal bronchioles and the alveoli. The 10 μm particles (PM_10_) have been widely studied in urban air pollution but PM_10_ from wildfires appear to have different effects on health than urban PM_10_. An 8-year study [350] investigating air pollution levels, including those from bushfires, and hospital admissions showed that a 10 μg/m^3^ increase in bushfire (but not urban) PM_10_ was associated with a 1.24 % increase in all respiratory admissions, a 3.80 % increase in COPD admission and a 5.02 % increase in adult asthma admissions. Increased levels of urban PM_10_ were associated with an increase in all-cause and cardiovascular mortality but not respiratory mortality alone.

Studies from Darwin, Australia, are particularly useful as there is very little background urban PM_10_ pollution and therefore most rises in air pollution in that area are secondary to bushfires [351]. Studies from this area [352, 353] report a significant increase in asthma and COPD presentations associated with raised PM_10_ levels from bushfire smoke.

The difference between urban and wildfire smoke is also illustrated in a study looking at the effect on macrophages exposed to wildfire and urban smoke in a murine model [354]. This showed that although cytokine production in response to wildfire smoke was lower than with urban derived particles, there was increased inflammatory (determined by measuring proinflammatory cytokines) and cytotoxic activity (as measured by biochemical markers of toxicity, apoptotic activity and nitrous oxide production) per cubic meter of air containing wildfire particles than with air containing only urban particulate matter. This was probably as a result of a higher concentration of PM_10_ particles in the wildfire smoke (10.3 μg/m^3^ compared with 5.5 μg/m^3^ in urban air). This increased concentration may have public health implications.

Wood smoke particles have also been shown to cause an inflammatory response in otherwise healthy humans. The Ghio et al., [355] study of 10 human volunteers exposed to woodfire smoke showed an increased level of blood neutrophils, and a neutrophilic influx into the lung evidenced in bronchial and bronchoalveolar lavage samples. Although this was a small study, the authors suggest that systemic and pulmonary inflammation in human subjects can result from exposure to wood smoke particles.

A crude extrapolated estimate from Finland suggested that high PM_2.5_ levels following wildfires in 2002 caused additional total mortality of 9–34 cases in a population of 3.4 million compared with what would normally be expected [356]. High levels of PM_10_ (both urban and from bushfires) were associated with a 1.8 % increase in ED attendances in a study in Victoria (carried out during a bushfire season in 2003–2003) [357]. A system of monitoring air pollution during and after wildfire events may provide useful public health information, facilitating preparedness for increased pressure on health care services, and should be considered after wildfire events. It would be important to monitor PM_2.5_ levels, not just PM_10_ levels. PM_10_ monitoring alone may not adequately represent the adverse effects of air quality that may be caused by the PM_2.5_ fraction [358].

### Exacerbations of respiratory conditions following exposure to wildfire smoke

A number of published studies have examined symptoms of chronic obstructive airway disease (COPD) and asthma following exposure to the products of combustion of wildfires. These may be summarized as follows.

Patients with COPD have been noted to be at increased risk as a result of wildfire – generated air pollution. A study [358] looking at symptoms of 21 local patients with COPD in the 2 months following the Denver wildfires of 2002 revealed that dyspnoea, cough, chest tightness, wheeze and sputum production all increased on days when PM_2.5_, PM_10_ and carbon monoxide levels in the atmosphere increased. This study provided a clear illustration of the link between air pollution from wildfires and COPD exacerbation. Wheeze was amongst the observed increased respiratory symptoms.

In a pediatric cohort study, 32 children with a history of wheeze suffered adverse effects from increased PM_10_ particles after the 2009 Australian bushfires. This study found that, although symptoms of evening wet cough worsened, dry cough and wheeze did not [359]. A study of 465 non-asthmatic teenagers affected by 2003 wildfires in Spain revealed that healthy patients who performed on the lowest quartile of lung function tests were more susceptible to the respiratory effects of wildfire smoke [360].

Data from the 1994 Sydney bushfires show that there was no increase in acute asthma related admissions in central Sydney [361] or Western Sydney [362] in the aftermath of the fires. This may not however reflect the true prevalence of asthma exacerbations, as only the more severe cases would be expected to present to the ED. In the days following the 1987 Californian bushfire, there was a 40 % increase in ED attendances [363].

It should be noted however that hospital attendance records may not be a reliable indicator of exacerbation of asthma attacks following exposure to wildfire smoke. This was revealed by a cohort study from Darwin (Australia) which showed that attendance studies may underestimate the respiratory symptoms [364]. In this study, 251 adults and children were asked to keep a record of their asthma symptoms during a 7 month bushfire period in 2004. During this time, PM_10_ ranged from 2.6–43.3 μg m^−3^. High PM_10_ levels were significantly associated with an onset of asthma symptoms, use of oral steroid medication, the mean daily symptom count and the mean daily dose of beta 2 agonists. Despite these indicators of an increase in asthmatic symptoms there was no recorded increase in the numbers of health care attendances or severe asthma attacks.

An increase in respiratory symptoms and deteriorating lung function was also seen in a study of findings reported by respiratory physicians and governmental reports in Indonesia at the time of the Indonesian wildfires of 1997 [365]. Worsening of respiratory symptoms were seen during the same period in surrounding countries such as Malaysia [366] and Singapore [367], illustrating the ability of particulate matter and air pollution to spread widely. 94 % of the air particles noted in Singapore in the haze following the Indonesian fires were PM_2.5_, and emergency department attendances related to the haze increased although overall hospital admissions due to respiratory effects did not [367]. These effects were observed more than 500 km from the bushfires. Delayed health effects may also occur. One study looking at health effects after the 2003 Canadian wildfires showed that there was a peak in respiratory consultations 5 weeks after the fires. This may be because of delayed respiratory health effects of wildfires smoke [166].

Following a peatbog wildfire in rural area of 42 North Carolina counties, an increase of cumulative risk of asthma, COPD, and pneumonia and acute bronchitis among the population of counties exposed compared to non-exposed counties was observed. ED visits of all respiratory diagnosis and cardiopulmonary symptoms were increased in the exposed counties [368].

### Uncertainty in the assessment of exposure to wildfire-related air pollutants

To evaluate the health impact of wildfire exposure, studies have used several exposure assessment methods [369] including self-reported questionnaires [370], routine data (number of wildfires) [371], air quality monitoring stations [357], and satellite data with visibility or aerosol optical depth (AOD) [368], etc. The purpose of these methods is to better estimate the personal exposure to wildfire emissions in order to better evaluate their health impact. Different degrees of uncertainty are associated with these methods [369].

Exposure assessments based on a self-administrated questionnaire which estimates the exposure to fire based on the declaration of the individuals threatened by flames are poor and underestimate real exposure. In addition, fire emissions may not be quantified but based on fire smell [370], which is not sufficient to determine an exact exposure. Several studies have also used air quality monitors to estimate the concentration of air pollutants emitted by a fire, especially PM. Limitations of monitoring stations include not being able to monitor an entire wildfire area and being damaged by the fire [372]. The veritable problem, however, is to distinguish wildfire emissions from those caused by other sources. This problem arises frequently for all quantitative methods.

More recently, satellite data are used to evaluate wildfire exposure because spatially-resolved particle mass data provided by satellites is superior to using data provided only by the nearest available monitoring station data [372]. However, it is important to recognize that errors in satellite resolution at the ground level may occur and satellite data cannot be retrieved (or has more uncertainty) if a region is cloudy or has strong reflection of the sun from bodies of water and over snow (ice and bright desert areas). In some regions, correspondence between satellite data (AOD) and PM_2.5_ has not been found, as may occur when aerosols are entirely aloft above the boundary layer and, therefore, do not influences surface air quality [373].

Notably, all of the described approaches may be combined within a modeling system, which is currently the only tool to allow separation of wildfires and other pollution sources impacts through different simulations. In addition, giving spatial coordinates with satellite data allows the estimating of wildfire exposure at zip code level, which may be not representative of individual exposure.

### What can be done to mitigate the potential asthmatic *sequelae* of exposure to wildfire smoke?

When there is a threat of wildfires due to extreme climatic conditions or an outbreak has occurred unexpectedly there are a number of measures that can be taken by health authorities to reduce the impact of the fire upon asthmatics [374, 375].

These include:

1. Limit exposure wherever possible.

Considering the potential toxicity of wood fire smoke, it is advisable to minimize exposure:

- Air quality reports should be checked. These may have the potential to be used in conjunction with surveillance of symptoms to understand health effects and their link with air pollution.
- Indoor air should be kept as unpolluted as possible by keeping windows and doors closed and shutting off external ventilation.

Identification of persons at risk and removal from the exposure hazard if possible or encourage sheltering. In cases of severe particulate hazards, filtration masks may be of value. Evidence for the effectiveness of masks, however, is limited. The avoidance of outdoor activities for those vulnerable to respiratory pathology should be recommended [374].

Access to homes, health care facilities and resources may be impeded.

- Systems should be in place to ensure delivery of medication and provisions to those who need them, especially vulnerable groups.
- People living in areas prone to wildfires may be advised to keep a stock of 5 days’ worth of non-perishable provisions and medications.
- Measures to maximize safety of routes to and from vulnerable areas should be in place
- Housing and evacuation routes in rural areas should be clearly signposted

2. Emergency services and GPs should be prepared for increasing numbers of patients attending with respiratory symptoms.

- Those with chronic respiratory illness may experience a worsening in their respiratory symptoms.
- There may be an increased incidence of mild respiratory symptoms amongst previously healthy individuals, which may require some medical treatment.
- Increased doses of anti-inflammatory and bronchodilator medication may be required. Stocks of drugs should be sufficient to accommodate for this.
- Provision of extra respiratory support in emergency facilities for patients with smoke induced respiratory distress should be considered. Measures that should be considered include non – invasive respiratory support such as continuous positive airway pressure support (CPAP) and the provision of a supply of portable emergency ventilators for severe cases of respiratory distress and failure. For patients with exacerbations of asthmatic symptoms at home the provision of electrically-powered oxygen concentrators which can provide a low pressure flow of oxygen up to around 5 l/min will be useful for emergency domicile management of respiratory distress leading to hypoxia.

### Conclusions

Inhalation of the products of combustion from wildfires has been shown to lead to exacerbation of a number of respiratory conditions and asthma in particular. A number of simple measures can help to mitigate these effects including simple public health advice on avoiding exposure and to encourage sheltering.

With the current global increase in wildfires and of destruction of forests by deliberate burning, all health care workers, particularly general practitioners, respiratory and emergency physicians need to understand more about the associated health risks and the requirements for early recognition of effects and treatment. More research is needed to evaluate long-term health effects from exposure to wildfires, and careful identification and follow up of those exposed could help in this process. Health care workers treating casualties from wildfires should be encouraged to publish case studies of health effects to increase the evidence base available to the international medical community dealing with wildfires. Long term, longitudinal studies looking at health effects in populations exposed to wildfires are needed.

## Violent conflict and asthma

This section explores the linkages between violent conflict (hereafter “conflict”) and asthma and looks at whether conflict (e.g., causes and consequences) has any potential impact on asthma prevalence and treatment. The section starts by outlining some of the common characteristics and trends of conflict and of the countries most affected by it. It then looks at the possible linkages between conflict and asthma in general terms. Lastly, it explores the linkages between conflict, access to health services, and asthma treatment. The section concludes that insufficient data on asthma prevalence in countries affected by conflict and of the linkages between conflict and asthma does not allow establishing any direct and solid correlation. More research is needed to understand the specific interaction between conflict and asthma.

### Violent conflict: an overview of trends

Despite an overall decline of intra- and inter-state conflict since the end of the cold war, approximately one third of the world’s population currently lives in about 30 countries considered affected by conflict and fragility, mostly in Africa, in the Middle East, Western and East Asia. Since 2012, an increasing intensity of internal conflict is associated with the volatile situations in North Africa and the Middle East [376–379].

These countries are often characterized by situations where the state or institutions lack the capacity, accountability, or legitimacy to carry out basic governance functions, including to deliver services, especially to the poor, and to mediate relations between communities and between citizens and the state [376–380].

Conflict affected and fragile countries present some of the greatest development challenges of our times. Conflict limits development prospects, as demonstrated by the fact that conflict affected low-income countries are furthest away from achieving the Millennium Development Goals and many belong to the group of the least developed countries [376]. Conflict threatens development gains, causes human rights violations, population displacement, the breakdown of institutions, of essential services, and of the infrastructure. Importantly, at times of conflict the trust between citizens and the State and among social groups often breaks, undermining the very pillars of a society [380].

Conflict also has macro-level impacts. These include a decline in state capacity associated with a shrinking revenue base and reduced public spending, and economic stagnation as a result of a fall in exports, hyper-inflation, exchange rate depreciation, disinvestment, and capital flight.

Recent changes in conflicts have introduced additional layers of complexity. Most contemporary conflicts are of protracted duration and/or recurrent, intra-state, fought by irregular armed groups, are fuelled by economic opportunities and ethnic rivalry, and are impacted upon by global illicit trades and networks. Violence increasingly takes place in settings with higher income and in contexts of increasing urbanization. It increasingly affects civilian and wealthier population groups [381]. These trends are further explained below.

#### From poor to higher income

Many conflict affected and fragile countries are low-income. The association of low economic indicators and conflict is in some instances quite high. It is estimated that the typical low-income developing country faces around a 14 % risk of civil war in any five-year period [382]. On the other hand, on average a country that experienced major violence over the period 1981–2005 has a poverty rate 21 % points higher than a country that has seen no violence [376]. The gap in poverty between countries affected by violent conflict and others is widening and projections suggest that an increasingly number of poor people will be concentrated in the future in conflict affected and fragile states [383]. Over half of the Iraqi, Palestinian and Sudanese population, for example, is estimated to be living in poverty or extreme poverty, compared to a 20–25 % regional average. This is gradually changing. Beginning with the Balkan and Caucasus wars in the 1990s, many conflicts now take place in settings with higher incomes and life expectancies. In addition, a variety of conflict settings may be present in a single country (e.g. Soudan, Democratic Republic of Congo) [381].

#### Conflict is recurrent and changes over time

Conflicts are not a one-time event; they are on-going and repeated. Re-occurrence of violence in contexts that have already experienced a conflict is high, with an estimated 50 % of relapse into conflict in the first post-conflict decade [376, 384]. In addition, conflict changes over time and different forms of violence interact with each other and with internal and external stresses, including environmental and other global factors (e.g. illicit traffics such as arms, drugs). In recent times conflict diffusion and conflict escalation in countries and regions previously considered relatively stable and with good performance across a range of governance and socio-economic indicators (e.g. Middle East and North Africa) creates a new set of challenges [378].

#### Conflict goes beyond borders

The consequences of conflict, including its economic impact, are not confined to a country. Neighboring countries and regions are affected by spillovers, including increased insecurity and criminal activity, population displacement (e.g. nearly 75 % of world’s refugees are hosted by neighboring countries), stress on local services, and losses from deteriorating regional trade, among others. A country making development advances, like Tanzania, for example, loses an estimated 0.7 % of GDP every year for each neighbor in conflict [376].

#### Conflict is costly and drains resources

The impact of conflict on the national, regional and global economy is high. The global economic impact of containing violence was estimated to be US$9.46 trillion in 2012 or 11 % of Gross World Product and nearly double the value of the world’s agricultural production. The total global expenditure for health was estimated to be 6.5 trillion in 2012. The cost the typical civil war is estimated at least $50 billion, or equivalent to more than 30 years of GDP growth for a medium-size developing country [384, 385]. If the world were to reduce its expenditure on violence by 50 % it could fund the additional amount required to achieve the Millennium Development Goals (e.g., $60bn) [379].

#### Conflict and environmental degradation

In regards to the linkages between conflict, climate change, and environmental degradation, whilst there is some evidence that environmental stress can increase the severity and duration of conflict, research suggests that the empirical foundation for a general causal relationship between climate change, environmental degradation and armed conflict is indicative [386–388]. Countries that are characterized by other conflict-promoting features are plausible candidates for climate-induced conflict. In the long term, this may change as climate change may increase risk of conflict through environmental displacement and heightened competition for resources.

#### Conflict trap

The interaction between the causes and consequences of conflict, including weak governance, low levels of human development, stagnant economies, high poverty rate, and various forms of inequality and exclusion push countries in a vicious cycle of violence, fragility, and economic decline, a *conflict trap*, from which it is very difficult to escape [382].

Taking into account the trends here highlighted, the next section explores potential linkages between conflict and asthma.

### Linkages between conflict and asthma

The limited availability of accurate socio-economic data, including on asthma prevalence, in most conflict affected and fragile countries, particularly in low income ones, does not allow identifying a clear and direct correlation between conflict and asthma. High death rate due to asthma recorded in a number of low-income conflict affected and fragile countries in Africa are likely related more to a lack of access to health services, low life expectancy, and an overall high mortality rate than to any particular impact of asthma [389]. General trends in asthma prevalence and some of the common triggers of asthma also do not suggest a clear and direct link between asthma and conflict.

Recent changes in conflicts characteristics, mentioned earlier, however, are profoundly changing the demographics and disease burden of conflict-affected populations. These combined with trends in asthma characteristics (e.g., rising prevalence in low and middle income countries, with documented disparities related to social and economic status, and a marked increase in Africa in recent years) may suggest a potential for greater interaction in the future [159, 381].

Current knowledge suggests the following potential indirect links between conflict and asthma.

#### Conflict, vulnerability, poverty, and asthma

Conflict, poverty, and vulnerability are closely associated. During conflict, key social indicators typically fall and a cycle of degradation and human vulnerability is set in motion [390]. Vulnerable population and individuals, including poor people, and in particular children and women, are disproportionally impacted. These groups experience a sudden and long lasting worsening of their living conditions, more acute poverty (e.g., due to loss of assets, income and access to markets, and as social service spending falls), greater exposure to hazards, including health hazards, and to various forms of deprivation, and increased levels of stress. To the extent that asthma may be more concentrated among people of lower socio-economic status given their higher exposure to suboptimal, unhealthy environmental conditions (e.g. physical, social, and psychological conditions) it may be that poor and vulnerable people in conflict situations may be more exposed to asthma or may see their capacity to access preventive and curative measures dramatically reduced [159].

#### Conflict, stress and asthma

Depression, trauma and emotional distress are common consequences of conflict and are much higher among war-affected populations than the general population. Between 30–70 % of people who have lived in war zones suffer from symptoms of post-traumatic stress disorder and depression [391]. These conditions can have an impact on people’s vulnerability to asthma as psychological factors are recognized to have a role by generating, precipitating or exacerbating asthma and by affecting a patient’s asthma control [159].

#### Conflict, population movements, and asthma

The displacement of people is a major social and economic consequence of conflict, in the short term as well as in post-conflict periods. By end 2012, 45.2 million people were forcibly displaced worldwide. Reflecting an increase in intra-state conflict, data shows an increasing number of internally displaced persons (i.e., some 28.8 million) and a decreasing refugee population (some 15.4 million) [392]. An increasing number of displaced people seek refuge in urban environments rather than in refugee camps, where they often live informally alongside residents and economic migrants, forming a type of mixed migration (e.g., Iraqis in Syria and Jordan, and Zimbabweans in South Africa) [381]. Conflict induced migration, including from rural to urban centers, displacement to densely populated areas (e.g., refugee camps), and migration to third countries may create conditions that increase people’s vulnerability, including vulnerability to asthma. Linkages between conflict induced migration and asthma may be direct, e.g. migration from rural to urban areas or from one country to another may involve exposure to a new set of pollutants and allergens as well as changes in housing conditions, diet and accessibility to medical services which may affect migrants’ health [159]. They may be indirect, for instance where migration worsens overall living conditions and reduces access to health services including in the receiving areas and population. In addition displacements of people have a high impact on receiving communities, institutions, and services, which may result in a worsening of socio-economic and environmental conditions for an even greater number of people.

Taking into account the potential linkages here highlighted, the next section explores the relation between conflict, health service delivery, and the treatment of asthma more in detail.

### Conflict, health services and asthma

Conflict is associated with poor service-related outcomes, including health outcomes. As delivery systems suffer, and there is a decline in quality, provision, and access, people’s health deteriorates, and a whole range of health needs becomes unattended. The impact of conflict on health and of failed health systems is often immediately visible in increased morbidity, mortality and disability [390, 393].

In most conflict affected and fragile situations - particularly in low-income ones – the common conditions that enable the State to deliver public services and people ability to access them are in many instances already weak before the conflict started. These include sound governance practices, effective central policy framework, adequate budgets, technical skills, capacity for planning, data collection, and coordination, security, and socio-economic and political conditions. Service delivery is often available only in urban areas, in certain regions within a country, and/or to some population groups. Often they are already overstretched addressing primary, survival-oriented health needs, and endemic diseases.

During and just after a conflict capacities and budgets of key service providers are further eroded, or diverted to other urgent priorities, skilled health professionals are dramatically reduced, treatments become unavailable, and systems may break down completely or become a target of conflicting parties, particularly in remote rural areas. The health sector poses particular challenges for service delivery at times of conflict, as health services are often highly transaction-intensive, difficult to monitor, and depend on determinants that lie outside the sector (e.g., water/sanitation, the mother’s education, nutrition) [390].

Populations affected by conflict experience severe public health consequences due to insecurity, poverty, vulnerability, displacement or issues of trust in and legitimacy of service providers that may prevent people from accessing services altogether. Poor and vulnerable people, whose use of and/or access to health care interventions is already lower than among richer groups, are particularly affected [394].

Based on this evidence, it is highly unlikely that in conflict affected situations diseases like asthma, which entail substantive education, awareness and monetary costs would receive due consideration and that capacity and resources would be available for diagnosis and treatment, even if the prevalence were to be high [159]. Furthermore, conflict and its consequences would likely increase most barriers to reducing the burden of asthma, including poverty and inadequate resources, low public health priority for asthma compared to other diseases, poor health care infrastructure, a tendency for care to be acute rather than long-term, difficulties in implementing guidelines developed in wealthier countries, limited availability of, and access to, medication due to cost and distribution problems, and lack of patient education, under-use of self-management and use of unproven therapies.

Things may change. As the profile of conflict-affected countries and populations changes, so does the burden of disease. Health policies and interventions will have to be revised. Whilst infectious diseases, pneumonia, neonatal disorders and endemic diarrhea are likely to continue to cause substantial mortality and morbidity in underserviced, insecure regions of sub-Saharan Africa and Asia and in conflict settings with low-income and low life expectancy, overcrowding- related epidemics (e.g., cholera, measles) might be arising less frequently than previously as less conflict affected populations are likely to live in camp settings. Non-infectious chronic diseases are becoming increasingly prominent in conflict settings because of improved recognition of their importance, possible increases in their prevalence in some long-term conflict affected areas, and because conflicts in middle-income countries, and increasingly in urban settings, affects different population groups [381].

### Conclusions

Violent conflict has multiple, long- and short-term impacts on physical, economic and social capital, development, and human well-being. The consequences of conflict are felt at various spatial levels, within the immediate area of conflict, in neighboring countries, and globally.

Current trends suggest a change in characteristics, patterns, and concentration of conflict with a shift towards intra-state conflict, middle-income countries, and urban environments. The spread of violence to countries with relatively good socio-economic indicators in the Middle East and North Africa is likely to further change the patterns of conflict and it consequences.

Available data and research suggest that, although no solid and direct link can be established between conflict and asthma, some potential indirect relations could be explored. These relate to the linkages between conflict, poverty and vulnerability, population displacement, conflict-induced stress, and asthma prevalence and trends.

The specific impact of conflict on health outcomes and access to health services is widely documented and has potentially a significant impact on availability of, and access to, diagnostic and treatment services for asthma. Given the changing patterns of conflict, also the dynamics between conflict and health and the provision of health services, including for asthma, are likely to change.

These trends, and trends in asthma prevalence, suggest a potential for increased interaction between conflict and asthma and the need to better understand the linkages between the two, which could be further explored in future research.

## Economical aspects of climate change

Asthma is a very common and increasingly widespread disease, in both western and developing countries, with significant health care costs that include direct costs, related to outflows during the disease period, and indirect costs, derived from missing work/school days and decreased productivity [395]. In the United States in 2009 the total costs attributed to asthma was about $20 billion dollars per year [396]. Moreover, the economic impact of asthma is higher in patients with more severe disease, and in patients with severe persistent disease a poor control is associated with higher costs (more than double) [397–399]. Indeed, asthma exacerbations have a demonstrated significant impact on health care and asthma-related costs [400]. In Europe, a recent population-based study in adults patients with persistent asthma from 11 European countries showed that the mean total cost per patient was EUR 1.583, ranging from EUR 509 in patients with controlled asthma to EUR 2.281 in patients with uncontrolled disease, and was largely driven by indirect costs; the expected total cost in the population aged 30–54 years of the 11 European countries was EUR 4.3 billion (EUR 19.3 billion when extended to the whole European population aged from 15 to 64 years) [401].

The economic impact of climate changes are just beginning to be understood. In the evaluation of climate change consequences, health costs have rarely been included, causing thereby substantial underestimation of related costs. Only few studies have assessed the future health costs associated to climate change [402, 403].

An important study evaluated the health-related costs from each type of extreme events that is recognized to be increasing as a consequence of changing climate: ozone pollution, heat waves, hurricanes, infectious disease outbreaks, river flooding, and wildfires. The authors used economic methods and state-collected or published data on the numbers of deaths, hospitalizations and emergency room visits to calculate the health cost of these six climate change-related events, that struck the United States of America between 2000 and 2009, and that resulted in 1689 early deaths, 8992 hospitalizations, 21,113 emergency department visits and 734,398 outpatient visits, and the total cost from all the six events. They found an estimated total cost that was more than $14 billion US dollars, with 95 % due to the value of lives lost prematurely [404].

In 2011 The Union of Concerned Scientists, a nonprofit partnership of scientists and citizens aimed at achieving practical environmental solutions in the United States of America, published a document in which the health care costs associated with ozone pollution, as a result of climate change, were estimated to be approximately $2.7 billion and $5.4 billion US dollars for an increase of ozone levels of 1 ppb and 2 ppb, respectively [405]. Regarding asthma, the health care costs of climate change are due to an increase in the number of patients and to an increase in disease severity.

Given that increases in temperature from climate change may increase ground-level ozone as well as aeroallergen concentrations, the interactions between these two airway noxious agents are very important [406]. Moreover, the close relationship between pollution, as a result of human activities, and global warming is now clearly demonstrated, and in patients with allergic and respiratory diseases the exposure to pollutants and the global warming act in a synergistic way in worsening the disease [407]. Specifically, ground-level concentration of ozone (O3), a potent oxidant, is likely to be related to climate change because it increases proportionally to the temperature [408]. Animal models and studies in humans have shown that ozone worsens bronchial inflammation associated with increased oxidative stress, and airway responsiveness; in asthmatic patients this results in acute respiratory symptoms and reduced lung function [409, 410]. Thus, the exposures to ground-level O3 is associated with emergency visits and hospitalizations for asthma and worse asthma control [410, 412].

It is reasonable to think that the negative impact of climate change on respiratory diseases results in an increase in costs for these diseases. The direct biological effect of pollutants and heat waves on respiratory system, the effect on allergenic plants and pollen distribution and also the fact that an increasing proportion of the population lives in urban areas, where is more exposed to pollutants, result in an increased incidence and a more severe asthma. These, as reported above, are associated with an increase of health care and asthma-related costs related to direct costs (hospital admission, emergency visits, physician visits, diagnostics and medication) and indirect costs (work absenteeism, school days lost, disability) [397–401]. At global level, this increased costs due to worsening of asthma have not yet been clearly defined.

An important research conducted in Southern California (Riverside and Long Beach) evaluated the economic impact of the air pollution-related burden of asthma in an area where O3 levels increased from 29 to 57 ppb between 1996 and 2004 [413]. The authors estimated the indirect and direct costs due to asthma-exacerbations linked with pollution and the costs of health care for asthma cases attributable to traffic-related pollution exposure. They concluded that the total additional asthma-specific cost each year that is due to air pollution was $18 million US dollars.

Regarding European Union (EU) countries, Bartlett et al., evaluated the economic impact of environment attributable childhood health outcome. The authors used a cost-of-illness approach to assess health care costs and the environment attributable fraction modeling to assess the percentage of diseases due to environmental exposure, focusing on costs in 27 EU members in the year 2008. Considering asthma, they concluded that environmental attributable costs of children suffering from asthma in European Union was $1.6 billion [414].

Cost-of-illness studies provide a measure of the potential economic burden of a disease, estimating the amount that could be saved or gained if the disease is eradicated [415]. For asthma and allergic diseases, preliminary prediction of cost-of-illness indicates that direct and indirect costs will significantly rise. Bielory et al., developed a simple Babesyan/supply chain economic model in order to draw parallels between climate changes and allergic diseases and asthma. Preliminary data showed a potential 30 % rise within the next decade in medical costs for allergic diseases and asthma as climate change impact continue to rise at the same rate [416].

Health adaptation costs, now defined as the costs of taking measures to reduce or to cope with additional impacts arising as a result of climate change, are of comparable importance. Adaptation measures in response to actual or expected climatic stimuli or their effects have in fact significant costs. These measures comprise all actions taken to reduce, prevent, or treat these additional cases of disease or death, including actions outside the health sector. Health adaptation costs include: costs of improving or modifying health protection systems (like vector surveillance systems or aeroallergen monitoring and forecasting), costs of introducing novel health interventions (heat-wave or other broader early warning systems), additional costs for meeting environmental and health regulatory standards (air quality and water quality, urban/settlement planning, building design and heating, ventilating and air-conditioning), costs of improving or modifying health systems infrastructure, occupational health costs (measures to prevent the adverse impacts on productivity of workers), costs of health research on reducing the impact of climate change (education, research) [188].

In 2007 the United Nation Framework Convention on Climate Change (UNFCCC) has estimated a cost of $4–12 billion/year in 2030 for adaptation in the health sector in order to prevent additional cases of common diseases due to climate change in developing countries [401, 417]. The World Bank (2010) estimates an average annual adaptation costs in the health sector for diarrhea and malaria prevention and treatment around $2 billion over the 40- year period 2010–50 [418]. More recently, UNFCCC has estimated that $73 billion US dollars per year will be needed for adaptation measures by 2030, including $5 billion spent directly by the health sector and over $25 billion by sectors influencing public health, such as water supply and sanitation [419].

Also for asthma, adaptation costs will be needed in order to reduce the impact on allergic people (e.g. aeroallergen monitoring, aeroallergen forecasting, allergenic plant management, planting practices and policies, urban/settlement planning, building design and heating, air-conditioning) [189] measures for preserving biodiversity. The balance between the cost of undertaking such measures of adaptation and the economic benefit that would result by reducing the impact on asthma and allergic diseases must be established.

## Final conclusion

Climate change affects the social and environmental determinants of health – clean air, safe drinking water, sufficient food, and secure shelter. The direct damage costs to health by the WHO is estimated to be between US$ 2–4 billion per year by the year 2030. Asthma is a heterogeneous disease that is strongly influenced by environmental factors. Many of these factors are influenced by meteorological events and climate change that vary in type and intensity across the world. To our knowledge there has been no systematic appraisal of how these different factors, which are related to weather and climate conditions, produce their varied effects on this disease, or the future influence of global climate change on environmental exposures.

Observational evidence indicates that recent regional changes in climate, particularly increases in temperature, have already affected a diverse set of physical and biological systems in many parts of the world. A rapid increase has been observed in the number of hot days and severe meteorological events witnessed across the globe. The overall topic of the effects of meteorological conditions on asthma is of global scope; this document addresses the range of these conditions and their effects, regionally and nationally. As the World Allergy Organization is focused on increasing awareness of the rising prevalence of asthma and allergic diseases worldwide as a global public health concern, this paper presents information on various meteorological aspects. This document aims to fill the knowledge gap in the form of a comprehensive overview of the available evidence. We have chosen those fields where meteorological or related influences are greatest. Areas with weak health infrastructure – mostly low and middle income regions – will be the least able to cope with this. Research to understand the impact of these factors of climate change on asthma and allergic diseases, and strategies and policies to reduce emissions of greenhouse gases and air pollution, are crucial.
